# Genome-wide identification of *StU-box* gene family and assessment of their expression in developmental stages of *Solanum tuberosum*

**DOI:** 10.1186/s43141-022-00306-7

**Published:** 2022-02-11

**Authors:** Zahra Hajibarat, Abbas Saidi, Mehrshad Zeinalabedini, Ahmad Mosuapour Gorji, Mohammad Reza Ghaffari, Vahid Shariati, Rahim Ahmadvand

**Affiliations:** 1 Department of Plant Sciences and Biotechnology, Faculty of Life Sciences and Biotechnology, Shahid Beheshti University, Tehran, Iran; 2 Department of Systems and Synthetic Biology, Agricultural Biotechnology Research Institute of Iran, Karaj, Iran; 3 Agricultural Research, Education and Extension Organization (AREEO), Karaj, Iran; 4 Department of Vegetable Research, Seed and Plant Improvement Institute (SPII), Agricultural Research, Education and Extension Organization (AREEO), Karaj, Iran; 5 NIGEB Genome Center, National Institute of Genetic Engineering and Biotechnology, Tehran, Iran

**Keywords:** *U*-*box* gene, *Solanum tuberosum*, Phylogenetic, TFBS

## Abstract

**Background:**

The Plant U-box (PUB), ubiquitin ligase gene, has a highly conserved domain in potato. However, little information is available about *U*-*box* genes in potato (*Solanum tuberosum*). In this study, 62 *U*-*box* genes were detected in the potato genome using bioinformatics methods. Further, motif analysis, gene structure, gene expression, TFBS, and synteny analysis were performed on the *U*-*box genes*.

**Results:**

Based on in silico analysis, most of StU-boxs included a U-box domain; however, some of them lacked harbored domain the ARM, Pkinase_Tyr, and other domains. Based on their phylogenetic relationships, the *StU*-*box* family members were categorized into four classes. Analysis of transcription factor binding sites (TFBS) in the promoter region of *StU*-*box* genes revealed that *StU*-*box* genes had the highest and the lowest number of TFBS in MYB and CSD, respectively. Moreover, based on in silico and gene expression data, variable frequencies of TFBS in *StU*-*box* genes could indicate that these genes control different developmental stages and are involved in complex regulatory mechanisms. The number of exons in *U*-*box* genes ranged from one to sixteen. For most *U*-*box* genes, the exon–intron compositions and conserved motifs composition in most proteins in each group were similar. The intron–exon patterns and the composition of conserved motifs validated the *U*-*box* genes phylogenetic classification. Based on the results of genome distribution, *StU*-*box* genes were distributed unevenly on the 12 *S*. *tuberosum* chromosomes. The results showed that gene duplication may possess a significant role in genome expansion of *S. tuberosum*. Furthermore, genome evolution of *S. tuberosum* was surveyed using identification of orthologous and paralogous. We identified 40 orthologous gene pairs between *S*. *tuberosum* with *Solanum lycopersicum*, *Oryza sativa*, *Triticum aestivum*, *Gossypium hirsutum*, *Zea maize*, *Coriaria mytifolia*, and *Arabidopsis thaliana* as well as eight duplicated genes (paralogous) in *S. tuberosum*. *StU*-*box 51* gene is one of the important gene among other StU-boxes in *S. tuberosum* under drought stress which was expressed in tuber and leaf under drought stress. Furthermore, *StU*-*box 51* gene has the highest expression levels in four tissue-specific (stem, root, leaf, and tuber) in potato as well as it had the highest number of TFBS in promoter region. Based on our results, StU-box 51 can introduce to researcher to utilize in breeding program and genetic engineering in potato.

**Conclusions:**

The results of this survey will be useful for further investigation of the probable role and molecular mechanisms of *U*-*box* genes in response to different stresses.

**Supplementary Information:**

The online version contains supplementary material available at 10.1186/s43141-022-00306-7.

## Background

Ubiquitination is an extremely conserved process in eukaryotes which is extensively implicated in various cellular processes namely cell cycle control, transcription, and the circadian clock [6]. This intracellular proteolysis is mediated mostly by the ubiquitin-26S-proteasome system. This system is a modification pathway of intracellular protein for cytosolic, membrane-localized, and nuclear proteins. The aberrant or truncated, active, and short-lived proteins from different cellular pathways are degraded and thereby regulate the protein loads of the cell [30]. The ubiquitination is mediated by a three-step enzymatic processes, a ubiquitin-activating enzyme (E1), ubiquitin-conjugating enzyme (E2), and a ubiquitin ligase (E3), recognizing the substrate [1, 11]. Degradation of proteins through ubiquitin pathway involves two distinct and continuous steps. In this system, the ubiquitin complex mediates the alteration of proteins through a collection of reactions that activate, transfer, and bind ubiquitin to cellular proteins, catalyzed by E1, E2, and E3 enzymes, respectively. First, an E1-ubiquitin thioester bond is constituted between C-terminal Gly carbocyl group of ubiquitin and the active site Cys of the E1 enzyme by ATP-dependent reaction. Second, the E1 transmit the activated ubiquitin to the Cys residue of the E2 enzyme to form an E2-ubiquitin thiester-linked intermediate by transesterification. Finally, E2 facilitates the attachment of ubiquitin molecule to the target protein in the presence of E3. E3 ligase acts a key role in protein ubiquitination as E3 can recognize target proteins for modification [39]. A single protein or a protein complex binds the ubiquitin reaction, which could be awarded by E3 enzyme.

E3 ligase recognizes the cellular proteins undergoing Ub conjugation, the main specificity factor in the ubiquitin (Ub)–proteasome pathway is the E3 enzyme. Therefore, E3 ligases belong to different gene family in plants. There are more than 1000 E3s in cells that join Ub to proteins in a highly regulated manner [13]. The E3 ligases are one of the most abundant family among all three enzymes and are grouped into various families based on their structure, function, and substrate specificity. The main classes of the E3 ubiquitin ligases are RING (Really Interesting New Gene), HECT (Homologous to E6-associated protein C terminus), CRL (Cullin-RING ligase), and U-box [31]. Ubiquitin E3 ligase had a U-box domain, family of proteins with motifs including 70 amino acids [3]. Most of the U-box proteins possess E3 ligase functions.

Previous studies have detected diverse functions of E3 ligases in *Arabidopsis*, banana, tomato, cotton, and rice [9, 17, 24, 42]. Based on cell death assay, *OsU*-*box* 51 gene is a negative regulator of cell death signaling [42]. In *Arabidopsis*, AtPUB9/18/19/44 were detected to disconnect ABA biosynthesis directly or via signal transduction. *AtPUB9* controls the transcription factor (TF) ABI3, ABI4, and ABI5 enhanced ABA sensitivity during seed germination [23]. Likewise, AtPUB18/19 also induce ABA hypersensitivity and therefore, negatively control the ABA [15]. AtPUB44 ubiquitinates the AAO3 (abscisic aldehyde oxidase 3) through proteasome and influences the ABA biosynthesis. Other studies have revealed that the U-box genes are upregulated under abiotic and biotic stresses in *Arabidopsis* and Nicotiana [15, 39].

In Brassica, ARC1, a novel U-box, is needed during refusal of self-incompatible pollen in pistil, ubiquitinates, and destruction of the S-receptor kinase [26]. Thus, the *U*-*box* gene family is considered a significant E3 ubiquitin ligase, affecting many plants signaling pathways and functions differently than other E3 enzyme classes. However, the evolution of the *U*-*box* genes in potato is largely unknown. Potato (*S. tuberosum*) is one of the most non-cereal food crop which is a vital food security crop for the worldwide population. Although, *U*-*box* gene may play significant roles in the development of potato however, up to now, the *U*-*box* gene of potato is infrequently surveyed. In this survey, the conserved domain analysis, evolutionary relevance, intron and exon patterns, chromosomal location, and analysis of expression profile were surveyed, providing a theoretical basis for the analysis of *U*-*box* gene functions.

## Methods

### Detection of U-box genes in S. tuberosum

Two techniques were utilized to detect potential *StU*-*box* genes in potato as explained earlier [29]. As the first technique, protein homology search with accessible U-box proteins from *Arabidopsis*, rice, and tomato were performed. The second technique included retrieving the U-box protein sequence using hidden Markov model (HMM) analysis, with the Pfam number PF04564 including typical U-box domain from the Pfam HMM library. The *A*. *thaliana* and rice protein sequences were taken from TAIR and RAP-DB databases, respectively. The known tomato U-box protein sequences were taken from NCBI, utilized as query sequences for tBLASTn program in potato to search for similar protein sequences. All putative sequences were approved with the SMART database and interproscan. The remaining 62 non-redundant candidates were recognized as StU-box proteins. The putative StU-boxs were validated by the presence of U-box, Armadillo (ARM), and protein tyrosine kinase (Pkinase_Tyr) (PF01545) using the hmmscan tool.

### Multiple sequence alignment and phylogenetic analysis

Sequence similarity analysis of StU-box proteins between *S. tuberosum*, *S. lycopersicum*, *G. hirsutum*, *O*. *sativa*, *Z. maize*, *C*. *mytifolia*, *T*. *aestivum*, and *A*. *thaliana* were utilized for multiple alignment as performed with MEGA 6.0 software. For phylogenetic tree construction, maximum likelihood method was utilized and its validation was done using multiple sequence alignments (CLUSTAL W with 1000 bootstrap replications) [27].

### Structural characteristics of U-box proteins

Peptide length, molecular weight, and isoelectric point (PI) were determined using the ProParam tool. MEME program and the Pfam tool were utilized to identify the conserved motifs and StU-box protein domains, respectively [4, 8]. Motif function was examined using the tool. GSDS program was utilized to analyze the exon-intron structures of *StU*-*box* genes.

### Chromosomal location and TFBS analysis

Chromosomal maps of *S*. *tuberosum U*-*box* genes were constructed by Chromosome Map Tools available at Mapchart. The up-stream 1500 bp of promoter regions of each *StU*-*box* genes were investigated using PlantPAN for the detection of Transcription factor binding sites (TFBS) in gene sequences.

### Synteny analysis and selective pressure estimation

To evaluate syntenic relationship, the orthologous genes between *S*. *tuberosum*, *S*. *lycopersicum*, *G*. *hirsutum*, *O*. *sativa*, *Z*. *maize*, *C*. *mytifolia*, *T*. *aestivum*, and *A*. *thaliana* were detected from Ensemble Plants. When the similarity exceeded 70%, it was considered to demonstrate orthologous genes. The paralogous genes in StU-box proteins were identified with similarity higher than 85% from Ensemble Plants. Orthologous and paralogous *StU*-*box* genes were visualized using Circos program. To categorize genes based on the selection type, the Ka/Ks was determined for each orthologous gene pair. Genes with Ka/Ks ratio < 1 indicated purifying selection, while the criterion for positive (adaptive) selection is Ka/Ks > 1.

### Gene expression analysis

#### Plant growth, tissue-specific and drought-induced expression profiles of StMTP genes

For tissue-specific expression analysis, 2-week-old seedlings were utilized to collect the roots, stems, and leaves, while 4-month-old seedlings were utilized to collect the tubers from the Seed and Plant Improvement Institute (SPII). For each genotype under drought stress, three potato tubers with 50 ± 10 g were planted in plastic bags (26 cm height, ~ 25 cm diameter) filled with soil. For drought-treatment expression analysis, two treatments were performed: drought stress and well watered (control). Each treatment had a randomized complete block design with three blocks (replications). For 6 weeks, all plants in both treatments were watered equally. Afterwards 6 weeks, plants (drought stress treatment) unwatered during 2 weeks, while the other plant (control treatment) was irrigated optimally. Sampling genotypes were performed in 2 weeks after drought stress. It means that sampling was performed in 8-week-old seedling.

The samples were collected from leaves and tubers of G29 genotype grown under mentioned conditions. Then, leaves and tubers under normal and drought (TN (tuber normal), LN (leaf normal), TS (tuber stress), and LS (leaf stress)) were quickly dipped into liquid nitrogen and stored at − 80 °C until RNA extraction.

#### RNA extraction and quantitative real-time PCR

Total RNA was extracted from tuber and leaf under normal and stress conditions after drought stress (6-week-old seedling) using RNA-Plus kit (*Sinaclone*) based on the manufacturer’s instructions. For the preparation of tissue-specific RNA, root, stem, leaf, and tubers were collected separately from 2-week-old seedlings. To remove residual genomic DNA contamination in RNA samples, DNase I (Fermentase Company) was utilized. The purity and concentration of RNA was determined by nanodrop as well as the quality of which was assessed using 1% agarose gel analysis. Then, cDNA synthesis was performed according to Easy cDNA Synthesis Kit instructions. Three replications were performed for the analysis of each gene. The potato EF-1α gene was utilized as reference gene. The gene-specific primers were designed using Vector NTI. Table S3 lists the primers and PCR conditions for amplification of StU-box 51, StU-box 27, StU-box 15, and StU-box 3, as well as the reference *EFα1* gene. Real-time was performed on ABI 7500 using SYBR Green Supermix as described in the producer’s guidelines. Analysis of gene expression was performed using the 2^-ΔΔCQ^ method for individual genes versus EFα1 as the internal control.

#### Gene ontology analysis of DEGs and protein-protein interaction of network analysis

Classification of DEGs by gene ontology (GO) analysis were performed using Blast2GO indicating probable pathways captured by genes involved in biological processes, molecular functions, and cellular component. The GO database generated an overview of the functional pathways in plant growth and developmental stages in potato**.** String (http://string-db.org/) was utilized to detect co-expressed genes and to draw the protein-protein interaction networks.

## Results

### Identification and characterization of U-box genes

In this survey, 62 genes were detected in the potato genome. The StU-box protein includes a 123 (StU-box 24) and 1484 (StU-box 32) aa U-box conserved domain. The molecular weight of StU-box was from 13781.97 kD (StU-box 24) to 166346.74 kD (StU-box 32). The PI was in the range of 5.01 (StU-box 45) to 9.18 (StU-box 35). Analysis of subcellular localization anticipated that 96% of the StU-box proteins were distributed in the nucleus and that only 8% were distributed in the cytoplasm (Table 1). These findings indicated that the majority of StU-boxs function in the nucleus.

**Table 1:** Characterization of StU-box genes in potato

Name genes	Classes	Groups	Number of amino acids	MW (kDa)	pI	Location
StU-box 1	Class I	U-box	1107	123,908.47	6.19	Nucleus
StU-box 2	Class I	U-box	415	45,438.52	6.21	Nucleus
StU-box 3	Class I	U-box	421	47,625.46	8.58	Nucleus
StU-box 4	Class I	U-box	451	50,425.96	8.79	Nucleus
StU-box 5	Class II	U-box and ARM	993	110,626.91	5.53	Cytoplasm, Nucleus
StU-box 6	Class I	U-box	411	45,845.54	8.43	Cell membrane, Nucleus
StU-box 7	Class I	U-box	454	51,351.98	8.30	Nucleus
StU-box 8	Class I	U-box	392	43,658.85	8.89	Nucleus
StU-box 9	Class I	U-box	406	45,325.95	8.92	Nucleus
StU-box 10	Class I	U-box	410	45,770.37	8.53	Nucleus
StU-box 11	Class I	U-box	406	45,383.30	9.00	Nucleus
StU-box 12	Class I	U-box	418	47,340.67	8.59	Nucleus
StU-box 13	Class I	U-box	407	45,417.16	8.92	Nucleus
StU-box 14	Class I	U-box	407	45,879.18	8.25	Nucleus
StU-box 15	Class II	U-box and ARM	892	100,112.52	5.79	Nucleus
StU-box 16	Class I	U-box	736	81,487.49	6.51	Nucleus
StU-box 17	Class IV	U-box and Ufd2p	1040	117,961.67	5.33	Nucleus
StU-box 18	Class II	U-box and ARM	486	53,465.24	6.85	Nucleus
StU-box 19	Class I	U-box	234	26,559.35	5.90	Cytoplasm
StU-box 20	Class I	U-box	1008	113,927.23	6.54	Nucleus
StU-box 21	Class I	U-box	735	81,319.01	6.19	Nucleus
StU-box 22	Class I	U-box	1007	112,311.90	5.38	Cytoplasm
StU-box 23	Class I	U-box	682	76,344.41	8.05	Nucleus
StU-box 24	Class I	U-box	123	13,781.97	6.58	Cell membrane, Nucleus
StU-box 25	Class II	U-box and ARM	535	58,830.95	8.63	Nucleus
StU-box 26	Class III	U-box and kinase	809	90,711.44	6.21	Nucleus
StU-box 27	Class IV	U-box and usp and kinase	787	88,990.19	5.78	Nucleus
StU-box 28	Class II	U-box and ARM	611	68,632.65	6.23	Nucleus
StU-box 29	Class I	U-box	647	69,896.18	8.44	Nucleus
StU-box 30	Class I	U-box	411	46,061.25	5.55	Nucleus
StU-box 31	Class I	U-box	443	49,617.63	6.06	Nucleus
StU-box 32	Class III	U-box and kinase	1484	166,346.74	5.75	Nucleus
StU-box 33	class I	U-box	418	46,061.50	8.80	Nucleus
StU-box 34	Class I	U-box	813	89,574.11	5.46	Nucleus
StU-box 35	Class I	U-box	442	50,230.38	9.18	Nucleus
StU-box 36	Class I	U-box	449	48,963.46	7.05	Cytoplasm, Nucleus
StU-box 37	Class I	U-box	404	45,725.64	5.66	Nucleus
StU-box 38	Class II	U-box and ARM	1046	117,247.83	5.86	Nucleus
StU-box 39	Class I	U-box	772	85,252.48	5.35	Nucleus
StU-box 40	Class II	U-box and ARM	692	76,220.66	8.77	Nucleus
StU-box 41	Class III	U-box and kinase	847	94,823.40	6.38	Nucleus
StU-box 42	Class II	U-box and ARM	650	70,580.83	7.85	Nucleus
StU-box 43	Class II	U-box and ARM	560	61,642.57	6.52	Nucleus,
StU-box 44	Class II	U-box and ARM	821	89,688.53	5.92	Nucleus
StU-box 45	Class I	U-box	428	46,916.19	5.01	Nucleus
StU-box 46	Class IV	U-box and Ufd2p	1019	115,679.95	5.17	Nucleus
StU-box 47	Class I	U-box	421	47,863.21	8.88	Nucleus
StU-box 48	Class II	U-box and ARM	661	72,292.86	5.54	Nucleus
StU-box 49	Class II	U-box and ARM	679	74,427.39	7.14	Nucleus
StU-box 50	Class IV	U-box and WD40	1040	116,153.41	5.62	Nucleus
StU-box 51	Class IV	U-box and WD40	1284	144,291.39	5.53	Nucleus
StU-box 52	Class IV	U-box and kinase and usp	745	83,678.15	6.29	Cell membrane, Chloroplast,Cytoplasm, Nucleus
StU-box 53	Class I	U-box	1015	111,985.43	5.60	Cytoplasm, Nucleus
StU-box 54	Class II	U-box and ARM	778	85,362.50	6.18	Nucleus
StU-box 55	Class I	U-box	449	49,970.89	8.87	Nucleus
StU-box 56	Class I	U-box	347	39,282.24	7.32	Nucleus
StU-box 57	Class I	U-box	420	46,905.47	8.76	Nucleus
StU-box 58	Class IV	U-box and kinase and usp	664	72,580.90	6.01	Nucleus
StU-box 59	Class I	U-box	404	45,641.31	8.92	Nucleus
StU-box 60	Class II	U-box and ARM	624	68,010.07	5.43	Nucleus
StU-box 61	Class III	U-box and kinase	557	63,507.13	6.08	Nucleus
StU-box 62	Class II	U-box and ARM	645	72,832.57	5.45	Cytoplasm, Nucleus

### Gene structure and motif analysis

**Fig. 1:**
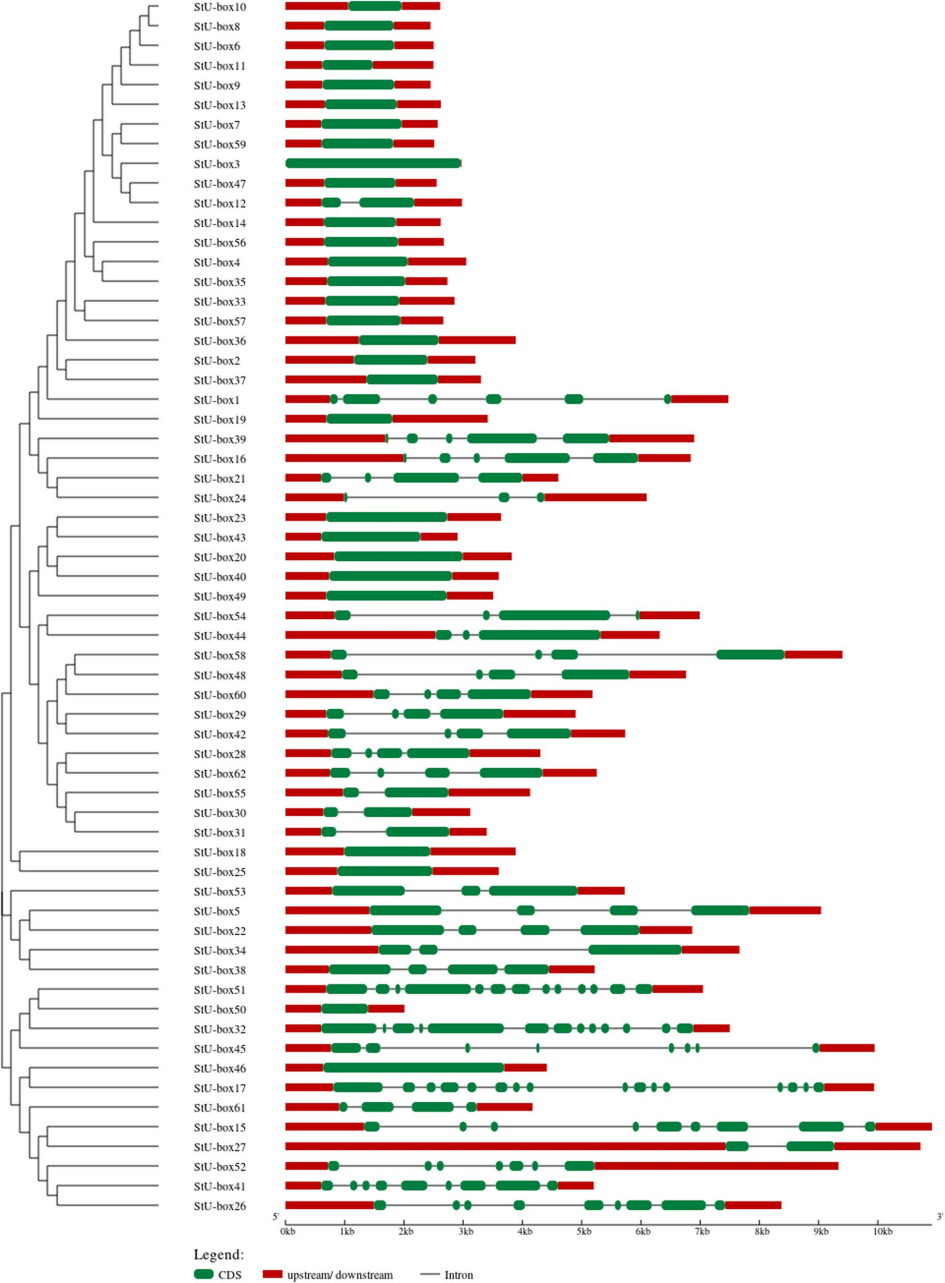
Distributions of the conserved domains in StU-box proteins

To gain more insight into the basic difference of *StU*-*box* genes, the exon-intron structure of each StU-box was examined. The number of exons in *StU*-*box* genes ranged from 1 to 16 (Fig. 1). About 61% of the class I genes possess no introns with approximately similar exon length, indicating genetic maintenance. A large number of introns was identified in class III and IV members with significant structural modifications. The 45% of all potato *U*-*box* genes family were characterized by only one exon, a sign of functional conservation among members of *U*-*box* gene family. Overall, our findings suggested that the ligase activity of *U*-*box* genes in potato is conserved. The structural organization also illustrated a relative amount of diversity among the members of *U*-*box* genes. The number of exons state the acquired assorted functional capabilities of the genes. The achievement of frequent exons and introns pattern could be a main outcome of the *U*-*box* genes expansion in potato.

**Fig. 2:**
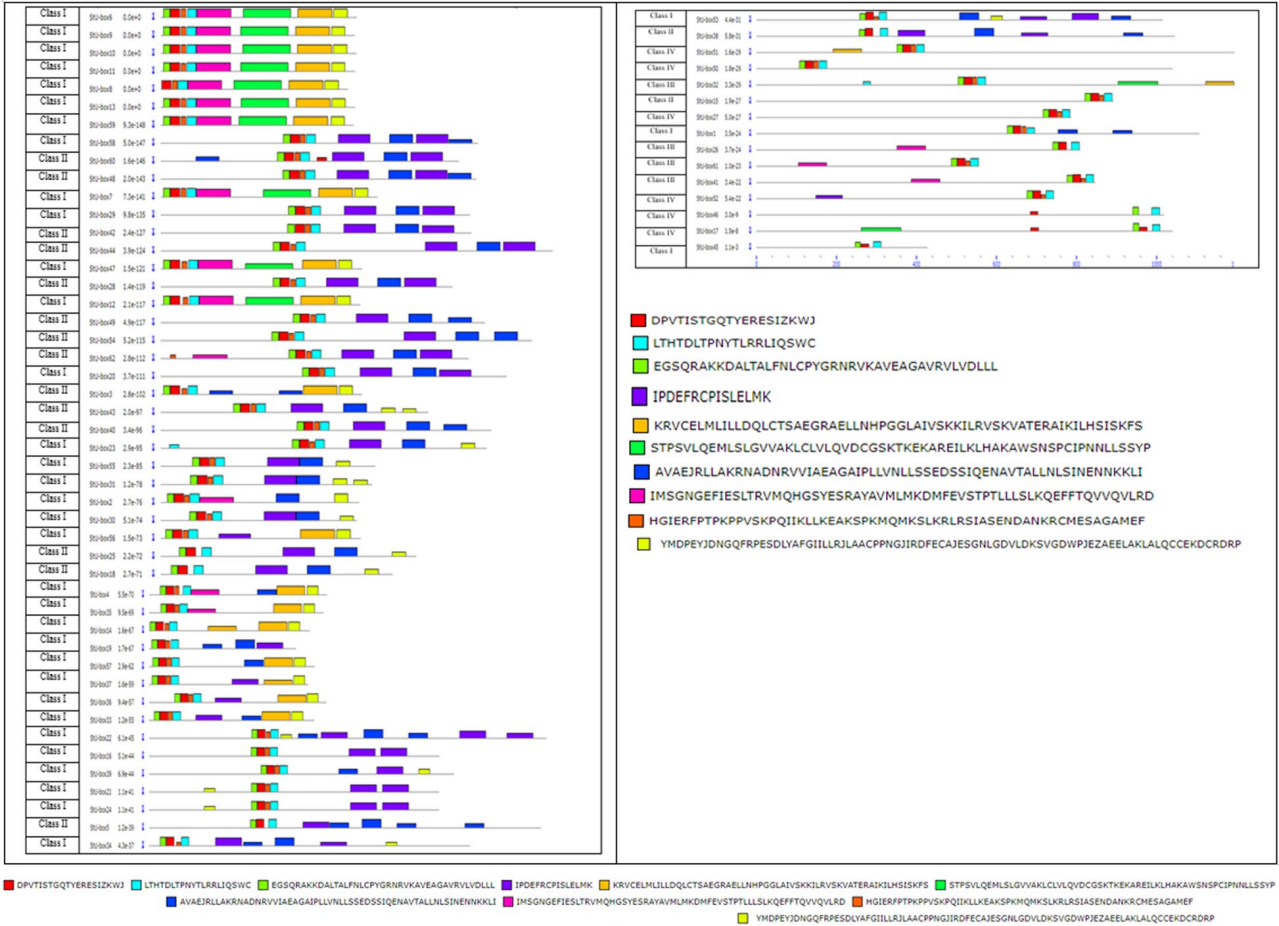
Conserved motifs, displayed in different colored boxes, as detected by MEME

Applying a two-component limited mixture model, all detected *U*-*box* genes were investigated for the presence of the original and ungapped motifs using MEME suite (Fig. 2). The structural diversity and the function of potato U-box proteins were anticipated; 10 preserved motifs in potato U-box were recognized using the MEME program. Motif 1 and 2 were present throughout the potato *U*-*box* members (Fig. 2; Table S1). Motifs 1 is conservative motifs in *U*-*box* genes; motifs 2 and 3 are conservative motifs in ARM; and motifs 4 is protein kinase motifs. The features of 10 motifs are revealed in Fig. 2, where motifs 5, 6, 7, 8, 9, and 10 are unidentified.

Motif 8 was widespread merely in class I and motif 9 was frequently existing in class I, whereas motifs 5 and 6 were characteristics of class I and II members which may minister separate biological functions. The symmetric and positional features of the recognized motifs consider not only the reservation of U-box domain functional facets but also the collection of further new domains over the progress of evolution. The detection of the 10 original motifs through the *U*-*box* genes provides indication for sharing biological functions. The common motifs patterns among the sequences are revealing of preserved evolutionary kinship and parallel cellular functions. Thus, it can be concluded that all the genes are implicated in the ubiquitin ligation.

### Chromosomal localization and phylogenetic analysis

**Fig. 3:**
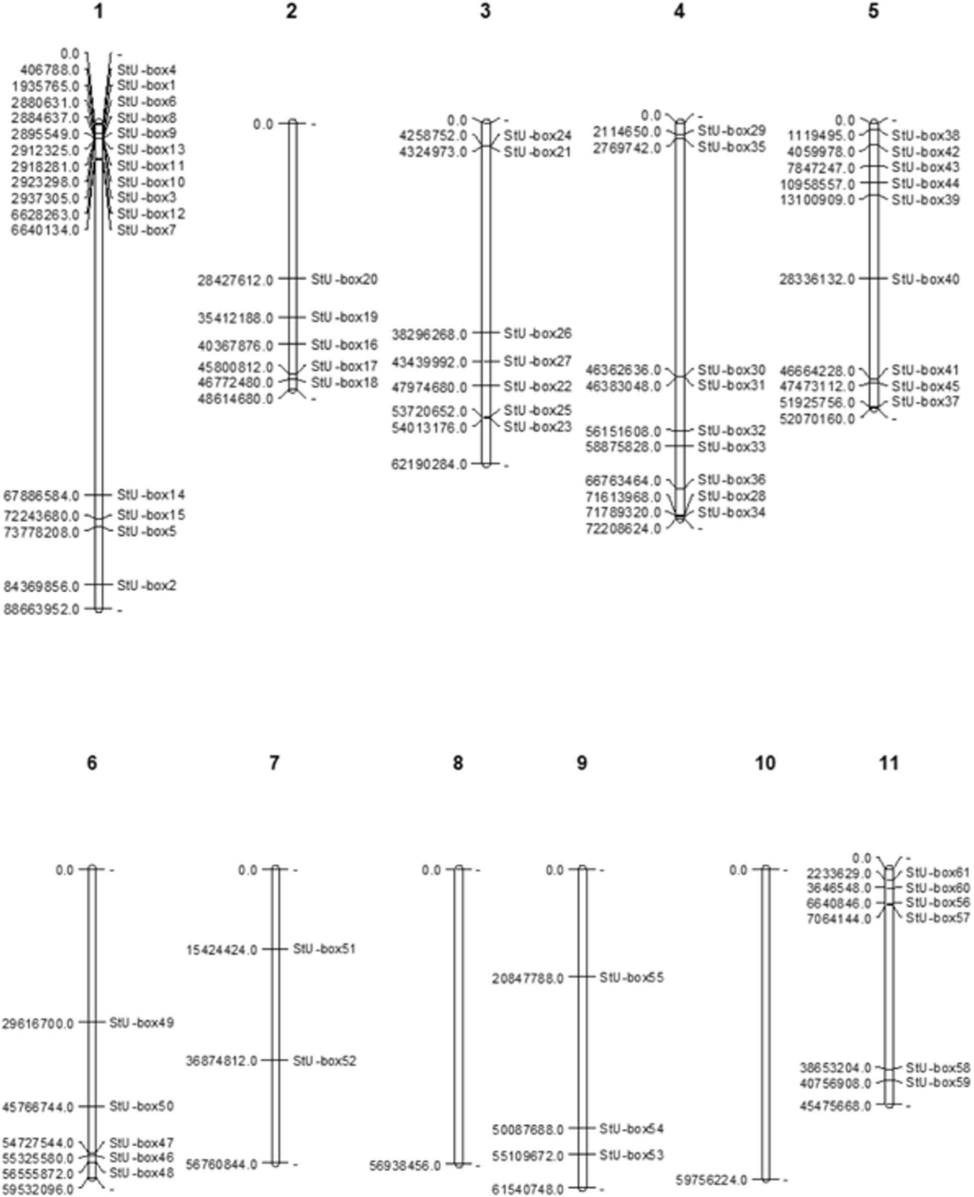
Distribution of *U*-*box* genes on *S. tuberosum* chromosomes

**Fig. 4:**
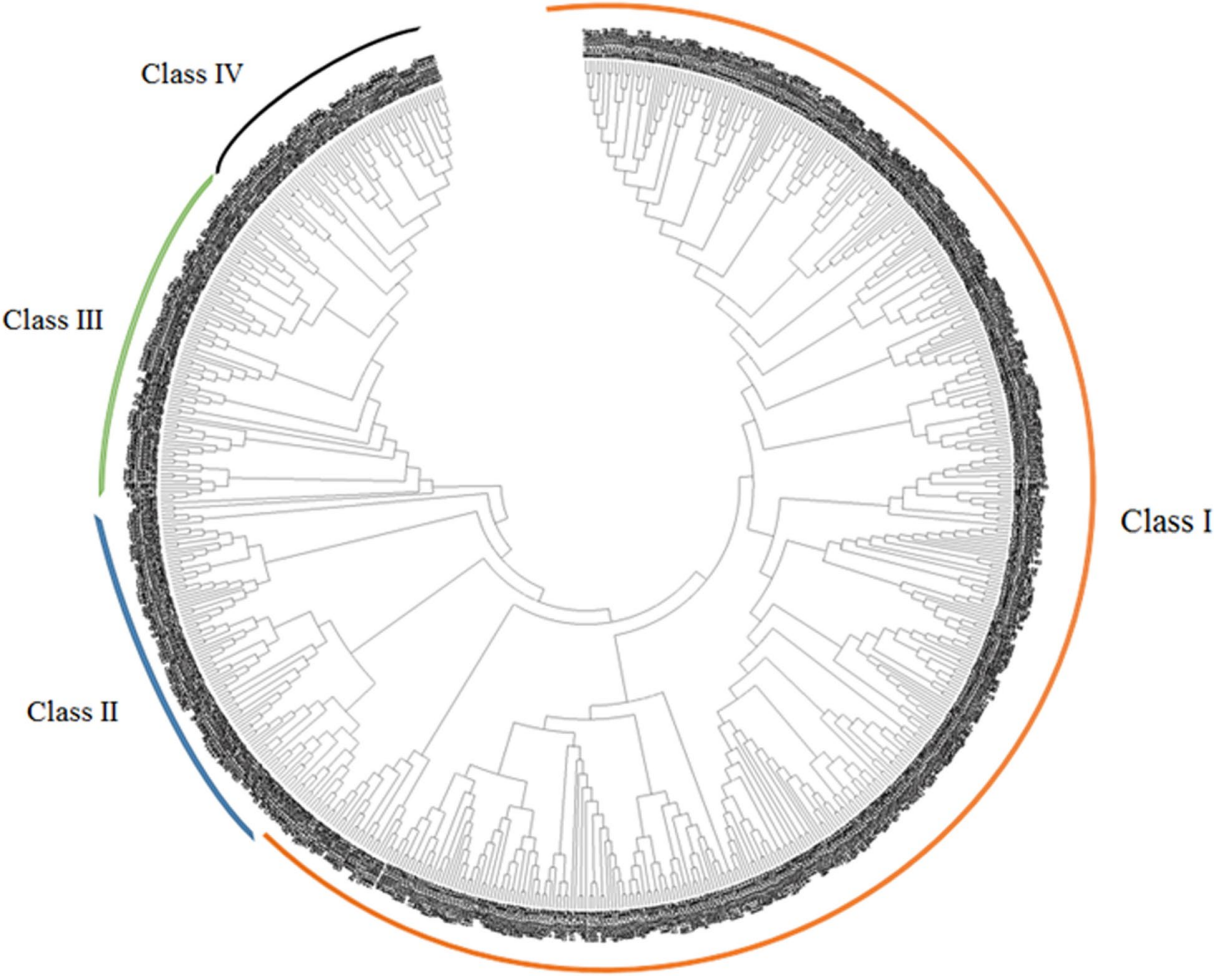
Phylogenetic relationships of U-box proteins in seven plant species (*S.tubersum* with *A*. *thaliana*, *S*. *lycopersicum*, *G*. *hirsutum*, *O*. *sativa*, *Z*. *maize*, *C*. *mytifolia*, and *T*. *aestivum*). The tree was constructed using the MEGA 6.0 software by the maximum likelihood method

The detected members of *U*-*box* genes were called StU-box 1 to StU-box 62 as per their chromosomal positions from chromosome 1 to 12 (Fig. 3). We have dispersed the U-boxes into four groups, based on the existence of the U-box domain (class I), U-box domain with armadillo repeats (class II), U-box domain with protein kinase domain (class III), and U-box domain with other domains such as WD40, KAP, Ufd2P, TPR, and RPW8 (class IV). To survey the evolutionary relations of *U*-*box* gene family members between potato and *Arabidopsis*, 62 U-box protein sequences from two species were carefully analyzed and a phylogenetic tress was constructed. The aa sequences of the U-box of 62 proteins from potato and 64 proteins from *Arabidopsis* were used. According to the classification of previous studies, 62 U-box proteins that were similar to the U-box in *Arabidopsis*, rice, cotton, wheat, citrus, tomato, and maize were categorized into four groups (class I, II, III, and IV). Phylogenetic analysis indicated that all detected U-box proteins from potato together with *Arabidopsis* were obviously divided into four subgroups. Of the four groups, class I possess the largest number of StU-boxs with 36 members. Four potato proteins, StU-box 26, StU-box 32, StU-box 41, and StU-box 61 were grouped in class III, and seven potato proteins were grouped in the class IV. StU-box 17 and StU-box 46 were clustered in class IV, containing the U-box and Ufd2p domains (Fig. 4). In the class II, 15 *StU*-*boxes* genes were clustered. Interestingly, these *StU*-*box* genes with similar genetic structures are grouped altogether. For example, StU-box 21/22/29 of class I each contained four exons, StU-box 26/41 of class III each contained eight exons, and StU-box 5/28/38/48/54/60/62 of class II each contained four exons.

### Analysis of the TFBS in the promoter regions of StU-box genes

TF binding sites (TFBS), regions of DNA binding sites in promoter, are important for transcription initiation of its target genes [36]. To detect the TFBS in the promoter regions, the 1000 bp upstream sequences of *StU*-*box* genes were retrieved from the database of *S. tuberosum* genome and analyzed using PlantPAN. As shown in the Table S2, 34 putative TFBS were detected in the promoter regions, the potential to regulate gene expression in response to environmental stresses, light response, tissue-specific response, other binding sites, and phyto-hormones. There are a number of diverse elements in the regulatory region of each corresponding gene and their diverse frequency in members of gene family. TFBS distribution in promoter regions of *StU*-*box* gene family is presented in the Table S2.

**Fig. 5:**
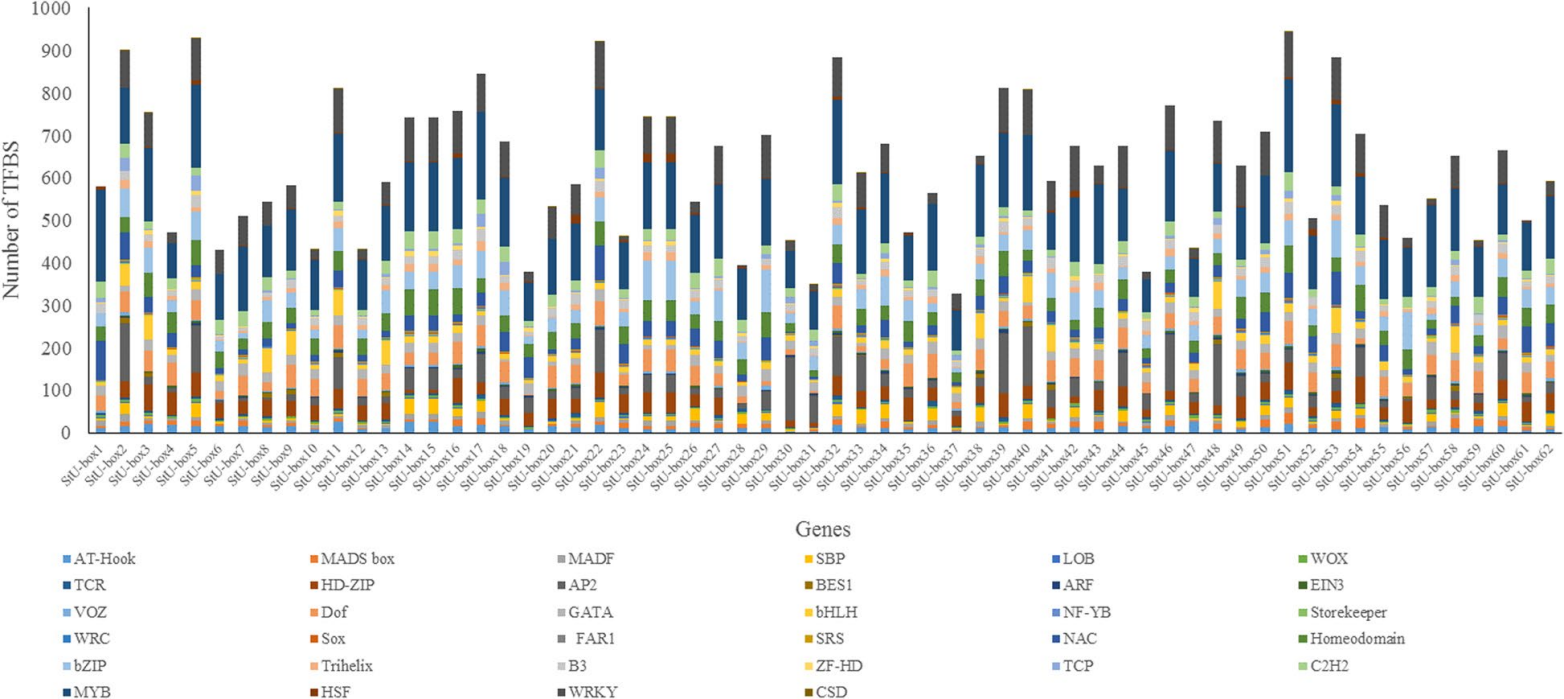
TFBS distribution in promoter regions of StU-box gene family

Among these common TFBS elements, MYB, WRKY, and AP2/ERF appeared to be the most frequent elements (with 8855, 3810, and 2776, respectively) and were commonly observed by all *StU*-*box* genes. Besides, three different types of members namely bHLH, Dof, and GATA were explored in light responsiveness elements. Further, five types of TFBS elements were found in response to hormone, namely AP2 involved in ethylene responsiveness, ARF in auxin responsiveness, EIN3 in ethylene and jasmonate responsiveness, VOZ in gibberellin responsiveness, and BES1 in strigolactone and Brassinosteroids responsiveness. Moreover, four types of TFBS elements involved in response to different environmental stresses include MYB for responsive to stress, WRKY for responsive to drought, HSF for responsive to cold shock and heat stress, and C2H2 response to abiotic and biotic stresses. Additionally, elements related to tissue expression contained AT-Hook for vasculature-specific expression, SBP for flower and fruit development, LOB for expression in root, MADS box and MADF for expression in floral organ, WOX for spatial and temporal expression, and TCR for male and female reproductive tissues. Furthermore, elements are related to transcription and expression namely NF-YB for embryo development, Storekeeper for plant-specific DNA-binding proteins and regulator of patatin expression, WRC for functions in DNA binding, and Sox for cell fate decisions during development. Notably, elements involved in stress control were distributed in the promoter regions of all *StU*-*box* genes, while elements involved in transcription and expression responsiveness were less abundant than the others (Table S2). It seems that the presence of these elements indicated that *StU*-*box* genes could be transcriptionally regulated by abiotic and biotic stresses (Fig. 5, Table S2). Results showed that *StU*-*box 51* and *StU*-*box 37* genes were the highest and the lowest number of TFBS in the promoter sequences, respectively.

### Orthologous and paralogous genes survey in StU-box

**Fig. 6:**
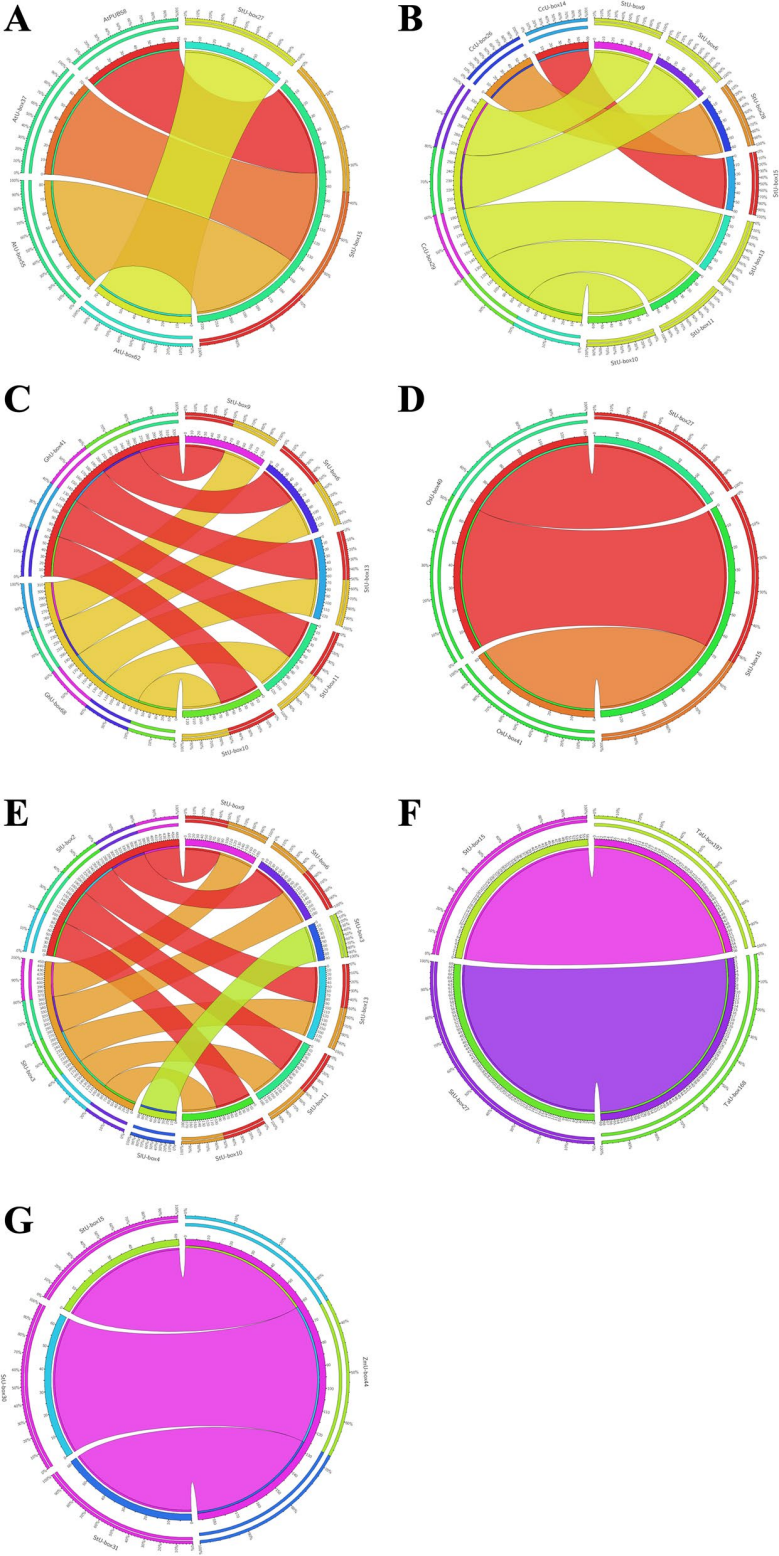
Synteny analysis of potato *U-box* genes with **A** *A*. *thaliana*, **B** *C*. *mytifolia*, **C** *G*. *hirsutum*, **D** *O*. *sativa*, **E** *S.lycopersicum*, **F** *T*. *aestivum*, and **G** *Z*. *maize* as visualized by online Circos

**Fig. 7:**
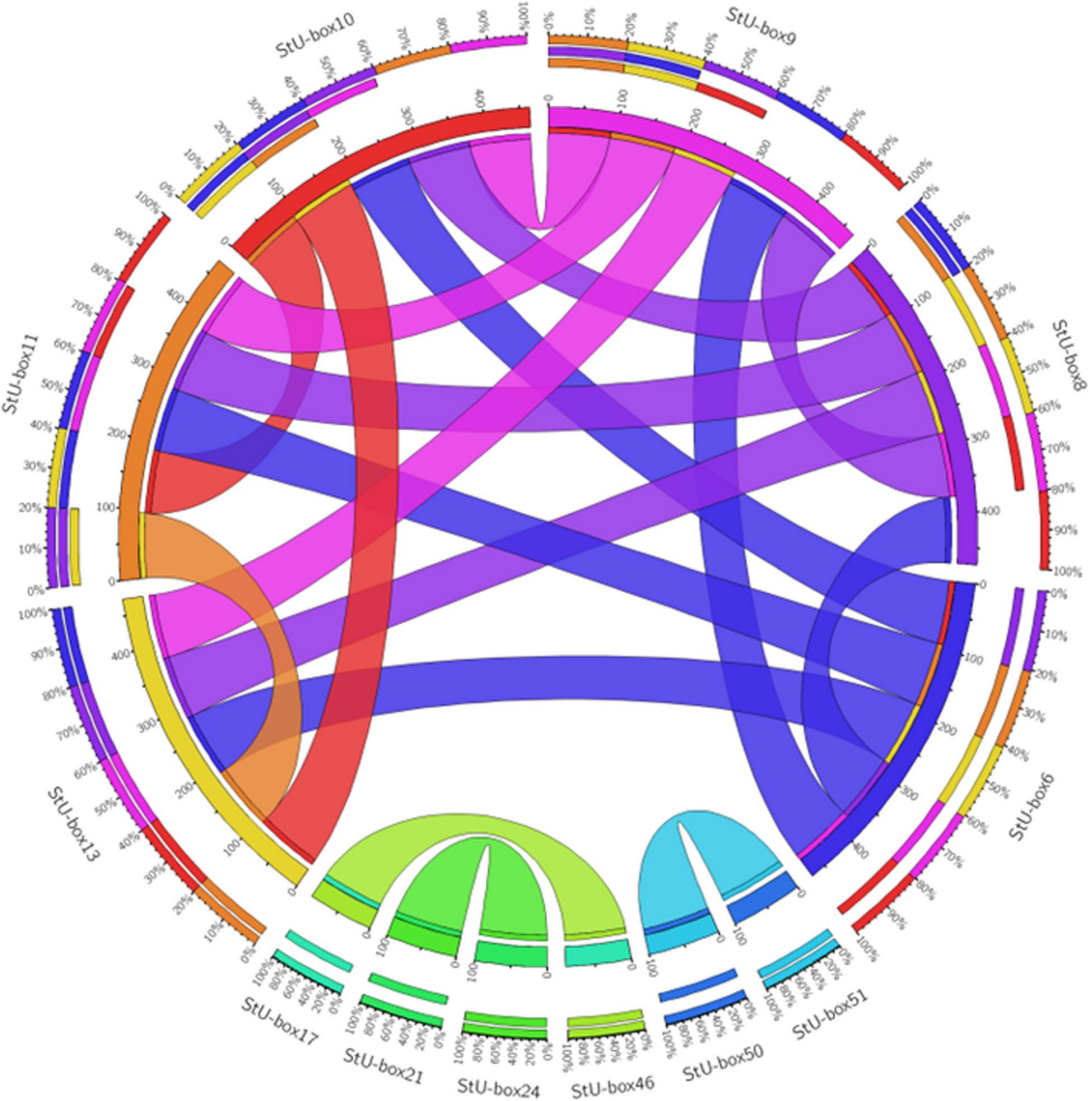
Synteny analysis of *StU-box* genes. Duplicated *StU*-*box* gene pairs (paralogous) relationship of *U*-*box* genes as visualized by online Circos

In this survey, evolutionary comparative analysis was done to detect orthologs of *StU*-*box* genes among *S. tuberosum* with *A. thaliana*, *S. lycopersicum*, *O. sativa*, and *T*. *aestivum* genomes. Based on our results, two genes in *S*. *tuberosum* revealed high similarity with four *Arabidopsis* genes and led to formation of four orthologous gene pairs. Further, one orthologous gene pairs was found in *S*. *tuberosum* with *T*. *aestivum* as well as with *O*. *sativa*. Eleven orthologous gene pairs were detected in *S*. *tuberosum* with *S*. *lycopersicum*. In the current survey, eight paralogous genes were identified. Orthologous genes between *S*. *tuberosum* with *A*. *thaliana* suggested that duplication plays a critical role in the expansion of *U*-*box* genes. In addition, eight paralogous gene pairs with identity more than 85% were detected in *U*-*box* gene family. These outcomes revealed that gene duplication may possess an important role in genome expansion. In the pathway, gene duplication included tandem/segmental duplications. Distribution of *U*-*box* genes on 12 chromosomes revealed that about 66.66% of *U*-*box* genes were implicated in tandem duplication with identity more than 90 percent (Figs. 6 and 7).

### Synteny analysis and gene duplication

We have observed that about 19.35% of the detected *U*-*box* genes participated in gene duplication occurrence in the *S. tuberosum* genome. Furthermore, tandem and segmental duplication were the key contributors to the expansion of potato U-boxes. Overall, both tandem/segmental duplications were detected. These segmental duplication contained four genes from the 12 genes, located on chromosomes 1 and 4. A total of twelve duplication events were recorded among the *U*-*box* gene family. The gene duplication was found on one or two loci. The synteny analysis showed that StU-box 6, 8, 9, 10, 11, and 13 were duplicated at two loci while residual candidates were observed at single locus.

To examine the selection types of the tandem and segmental duplication related to potato U-box genes, the synonymous (*Ks*) and non-synonymous substitutions (*Ka*) between the gene pairs were examined. *Ka/Ks* ratio less than 1 indicates purifying selection on the gene pairs, *Ka/Ks* = 1 indicates neutral selection, and *Ka/Ks* ratio more than 1 indicates positive selection on the gene pairs. A summary of the Ka/Ks ratios for the four tandem and eight segmental duplications are shown in Table 2. The detection of the nature of duplication and evolutionary pattern in the genome were determined using the Ka/Ks ratio [36]. Among the 62 StU-box members, we selected 12 pairs of duplicated blocks in the potato genome. Eight of the duplicated *U*-*box* genes in potato revealed a Ka/Ks ratio of less than 1, indicating that these one-to-one genes underwent purifying selection. StU-box 6/9, StU-box 7/12, StU-box 11/13, and StU-box 28/34 had a Ka/Ks ratio of more than 1, indicating that positive selection shaped these one-to-one genes. Most gene pairs of *Arabidopsis* and citrus underwent purify (negative) selection whereas, most genes of tomato, cotton, rice, wheat, and maize were subjected to positive selection. Our findings showed that tandem duplication occurred in four gene pairs. Taken all together, the outcomes indicated that the tandem and segmental duplications, as a leading component for the *U*-*box* genes extension, could efficiently contribute to the protection of the structures and functions of the genes. It can also be a cause behind the acquisition of novel functional domains on the genes [33].

**Table 2:** Ka/Ks analysis for *StU*-*box* genes

Duplicated pair	Duplicate type	Ka	Ks	Ka/Ks	Positive selection
StU-box 3/10	Tandem	2.9689	2.2984	0.7742	No
StU-box 3/11	Tandem	2.8414	2.2924	0.8068	No
StU-box 6/8	Tandem	3.5823	2.0766	0.5797	No
StU-box 6/9	Tandem	2.01	1.8276	1.103	Yes
StU-box 8/9	Tandem	2.5007	1.8512	0.7403	No
StU-box 7/12	Segment	3.2122	2.87	1.118	Yes
StU-box 10/11	Tandem	2.9291	2.451	0.8367	No
StU-box 13/10	Tandem	2.9633	2.5061	0.8457	No
StU-box 13/11	Segment	4.9234	2.3356	2.108	Yes
StU-box 21/24	Tandem	2.7183	2.3542	0.8661	No
StU-box 28/34	Segment	2.7192	2.946	1.0834	Yes
StU-box 30/31	Segment	2.3015	1.8702	0.8126	No
AtPUB1/2	Tandem	1.8911	3.4452	0.5489	No
AtPUB2/3	Tandem	1.8561	3.2687	0.5678	No
AtPUB3/1	Tandem	2.2704	3.1686	0.7165	No
AtPUB13/14	Tandem	3.2942	2.4233	1.3594	Yes
AtPUB27/28	Tandem	2.5159	2.6878	0.9361	No
AtPUB39/40	Tandem	2.2177	2.3145	0.958	No
AtPUB49/50	Tandem	2.6139	2.5701	1.017	Yes
AtPUB50/51	Tandem	2.6399	2.867	0.9208	No
AtPUB49/51	Tandem	3.8226	3.0968	1.2344	Yes
AtPUB57/58	Tandem	4.9102	4.8888	1.004	Yes
AtPUB63/64	segment	1.91	3.6343	0.5255	No
CcU-box 8/9	Tandem	2.4468	2.9408	0.832	No
CcU-box 13/14	Tandem	2.5589	2.7006	0.9475	No
CcU-box 18/19	Segment	2.1265	2.7881	0.7627	Yes
CcU-box 26/27	Tandem	3.6119	3.8546	0.937	No
CcU-box 29/30	Tandem	2.5775	2.786	0.9251	No
CcU-box 43/44	Tandem	4.824	2.6361	1.83	Yes
GhU-box 5/6	Tandem	3.3199	3.084	1.0765	Yes
GhU-box 27/28	Tandem	0.7533	0.749	1.006	Yes
GhU-box 39/40	Tandem	3.094	3.1003	0.997	No
GhU-box 41/42	Tandem	2.006	2.125	0.944	No
GhU-box 44/45	Tandem	2.9385	2.5408	1.1566	Yes
GhU-box 49/50	segment	2.4743	2.3595	1.0487	Yes
GhU-box 60/61	Tandem	2.2918	2.2	1.041	Yes
GhU-box 68/69	Tandem	1.789	1.4728	1.214	Yes
GhU-box 92/93	segment	2.6536	2.5873	1.0256	Yes
OsU-box 11/12	Tandem	1.7712	1.12	1.58	Yes
OsU-box 44/45	Tandem	1.8283	1.7828	1.0255	Yes
OsU-box 40/41	Tandem	2.588	3.8332	0.6752	No
OsU-box 62/63	Tandem	2.6172	4.7782	0.5477	No
OsU-box 61/63	Tandem	0.7552	0.698	1.081	Yes
OsU-box 61/62	Tandem	3.3893	3.3116	1.0235	Yes
OsU-box 63/64	Tandem	2.2926	2.1725	1.0553	Yes
OsU-box 57/58	Tandem	4.4363	3.561	1.246	Yes
OsU-box 67/68	Tandem	2.7202	2.9911	0.9094	No
SlU-box 57/58	Tandem	2.8944	1.6806	1.7222	Yes
SlU-box 31/32	Tandem	2.3652	2.6092	0.9065	No
SlU-box 2/3	Segment	0.071	0.0499	1.423	Yes
SlU-box 2/4	Tandem	3.0671	2.3017	1.3325	Yes
SlU-box 2/5	Tandem	2.4453	2.1782	1.1226	Yes
SlU-box 3/4	Tandem	3.0011	2.4993	1.2008	Yes
SlU-box 3/5	Tandem	2.7222	1.1462	2.3749	Yes
SlU-box 4/5	Tandem	2.1477	2.1559	0.9962	No
SlU-box 6/7	Segment	1.6731	2.1477	0.779	Yes
TaU-box 70/71	Tandem	3.3179	3.3289	0.996	No
TaU-box 96/97	Tandem	3.009	3.015	0.998	No
TaU-box 107/108	Tandem	2.91	2.4663	1.1799	Yes
TaU-box 116/117	Tandem	4.2555	3.6081	1.1794	Yes
TaU-box 122/123	Tandem	4.67	4.5236	1.0332	Yes
TaU-box 154/155	Tandem	3.2301	3.178	1.02	Yes
TaU-box 168/169	Tandem	3.5713	3.441	1.038	Yes
TaU-box 168/170	Tandem	4.6876	4.598	1.019	Yes
TaU-box 169/170	Segment	3.1908	3.1286	1.019	Yes
TaU-box 197/198	Segment	3.1082	2.7676	1.121	Yes
ZmU-box 18/19	Tandem	2.06	1.89	1.08	Yes
ZmU-box 43/44	Segment	1.98	1.75	1.13	Yes

Ka/Ks < 1 means negative selection, Ka/Ks = 1 means neutral selection, and Ka/Ks > 1 means positive selection

These outcomes indicate that basically, segmental duplications, but not tandem duplications, have contributed to the expansion of the StU-box in potato. Furthermore, the duplicated gene pairs have evolved mainly under the effects of purifying selection with no functional divergence after segmental duplications. Overall, tandem duplication indicated a very high Ka/Ks ratio [41]. Since tandem duplication has generally resulted in gene clusters in genome, these outcomes also indicate that genes within each cluster have evolved faster than others. Thus, this type of duplication would be more likely to produce new functions during the extended evolutionary history of the potato. In contrast, genome-wide duplication was characterized by very low Ka/Ks ratios (Ka/Ks < 1), showing that most of the genes in this category have retained their original functions during evolution. Our results are also in disagreement with previous reported study on *U*-*box* genes in potato [24] (Table 2).

### Analysis of gene expression of StU-boxs

#### The expression patterns of StU-box in tissue-specific

**Fig. 8:**
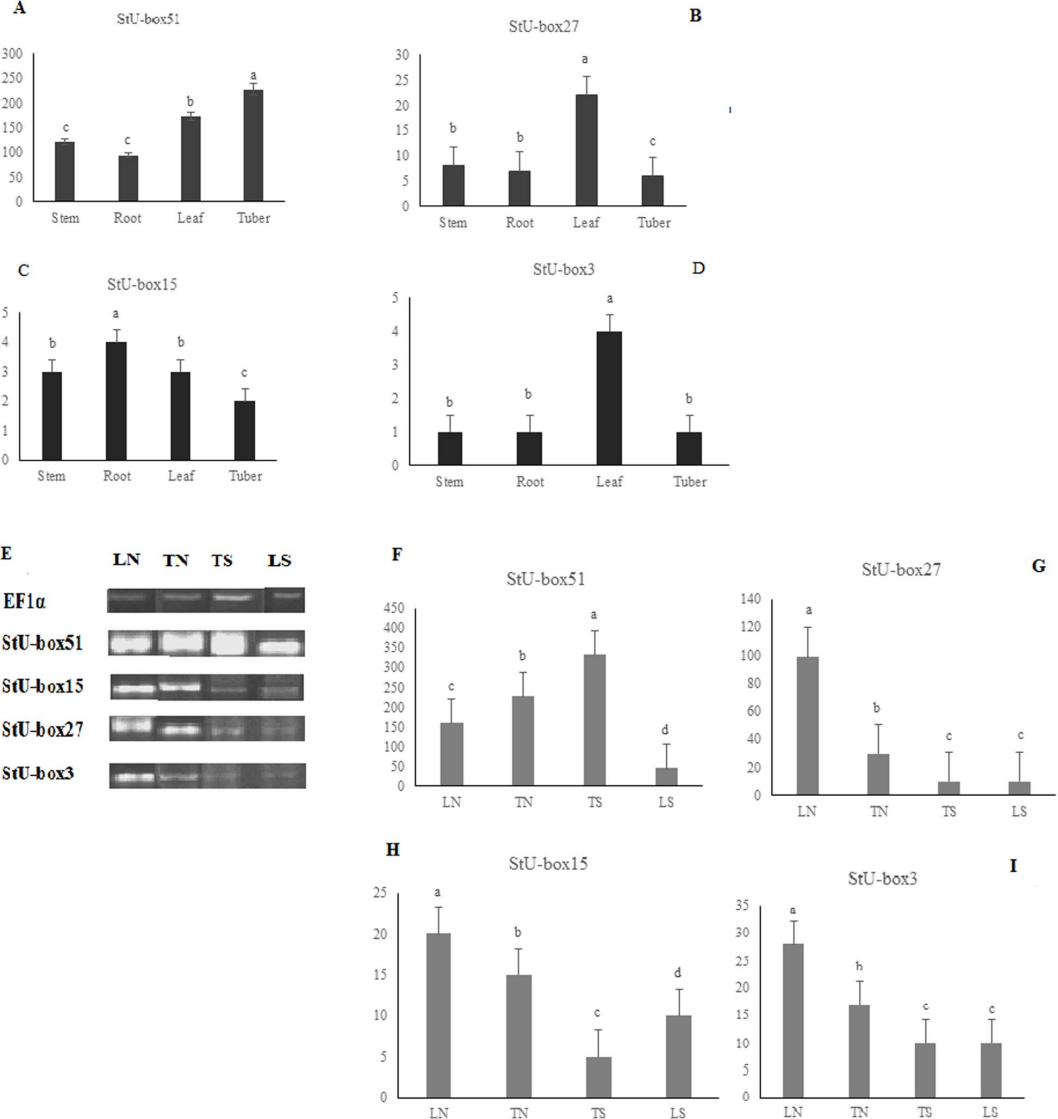
The expression patterns of four candidate genes (StU-box 51, StU-box 15, StU-box 27, and StU-box 3) in four tissues (tuber, leaf, stem, and root) in *S*. *tuberosum* in **A**, **B**, **C**, and **D** in tissue-specific, respectively. Also, **E** RT-PCR analysis of gene expression and **F**–**I** qRT-PCR analysis of four genes in tuber and leaf under drought and normal conditions; LN (leaf normal), TN (tuber normal), TS (tuber drought stress), LS (leaf drought stress). Error bars indicate the standard errors (SE) of average results

To further analyze the characteristics and function of the *StU*-*box* genes, the tissue-specific expression of the four *U*-*box* gene (StU-box 51, StU-box 27, StU-box 15, and StU-box 3) was analyzed. The expression pattern of *StU*-*box* genes in four different potato tissues, containing tuber, root, leaf, and stem were investigated using the qPCR. As shown in Fig. 8A, B, C, D, the tissue expression patterns of StU-boxs among the four genes were different. The expression levels of *StU*-*box 3* and *15* approximately were similar, although *StU*-*box 15* revealed higher expression than *StU*-*box 3* in four tissues. Also, StU-box 51 had the highest expression levels in tuber, leaf, root, and stem while StU-box 3 and StU-box 15 had the lowest expression levels in tuber, leaf, root, and stem. StU-box 27 possesses the maximum expression level in leaf, while it has the minimum expression in tuber, root, and stem. StU-box 51 displayed relatively higher expression levels than StU-box 27, StU-box 15, and StU-box 3 in the leaf, root, tuber, and stem. In *StU*-*box 3*, gene expression levels in leaf were high, whereas gene expression levels were low in root, stem, and tuber.

#### The expression patterns of StU-box under drought stress

To further understand the expression levels of *StU*-*box* genes influenced by drought stress, we selected four *StU*-*box* genes after investigating the structure, phylogenetic analysis, and examining their relative expression profiles by qPCR in leaves and tubers after drought treatment. The expression levels of drought and normal treatment were given in Fig. 8E, F, G, and H. The quantitative real-time PCR (qRT-PCR) used in this study are provided in Table S3. The *StU*-*box* genes showed variation in expression with dehydration stress, as compared to control (Fig. 8I). The results of qPCR analysis revealed that the StU-box 51 had the highest expression level in leaf and tuber (normal) under normal treatment while it was downregulated under drought condition in leaf. However, StU-box 51 was upregulated under drought stress in tuber. Furthermore, the expression levels of StU-box 51 was higher than StU-box 3, StU-box 27, and StU-box 15 in both leaf and tuber (normal and drought conditions) (Fig. 8E, F, G, and H). StU-box 27 had higher expression level in leaf as compared to tuber under normal condition, whereas it had lower expression level in tuber and leaf under drought stress. In addition, gene expression profile for StU-box 15 and StU-box 3 were nearly equal in leaf and tuber under normal and drought stress conditions. The expression of both genes were up-regulated under normal leaf condition but were downregulated in leaf and tuber under stress treatment.

#### Co-expressed gene network and GO analysis

**Fig. 9:**
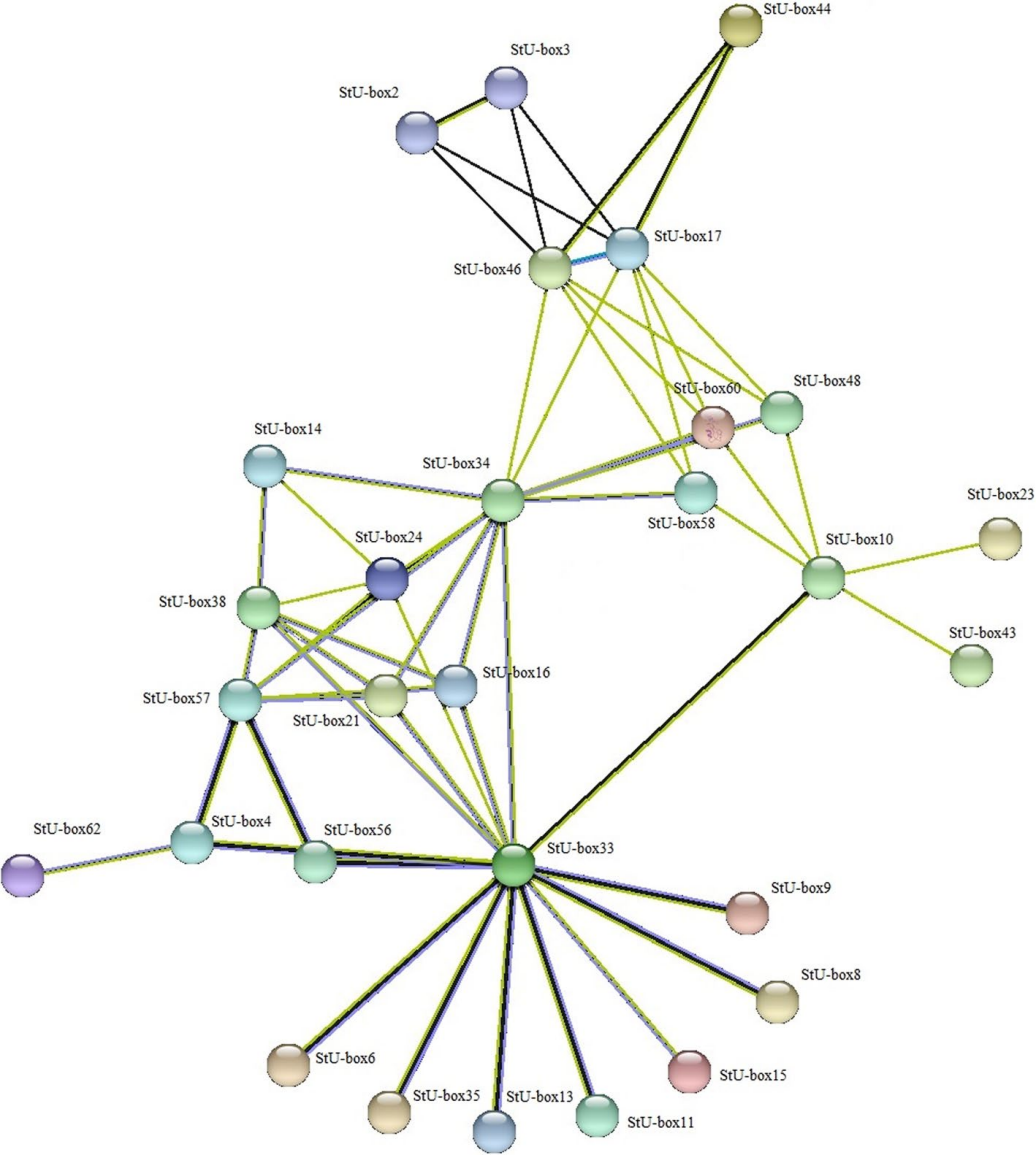
Interactions of co-expressed network genes of U-boxs in potato. The web based tool “String” (http://string-db.org/) was utilized to predict the interactions

The protein-protein network interaction of 62 genes revealed that most genes in the network were included in the class I of U-box. In this network, PGSC0003DMG400000791 (Stubox33) seemed to be the central protein involving in the pathway protein ubiquitination. PGSC0003DMG400000043 (StU-box 15) possesses an essential role in protein ubiquitination and protein modification. OsU-box 40 (upregulated under salt and against pathogen invasion) was found to be orthologous with StU-box 15. PGSC0003DMG400015790 (StU-box 27), another gene in this network, acts as a receptor protein kinase, trigging a defense response under abiotic and biotic stress conditions (Fig. 9). This receptor can mediate response to organic chemicals, namely the ethylene, cytokinin, and ABA hormones. For generation of defense responses, the activation of signal transduction cascades and protein ubiquitination are necessary for the modulation of plant immunity. On the other hand, some genes of U-box are involved in cellular regulation in eukaryotes, controlling a wide range of processes containing embryogenesis, hormone signaling, and senescence. StU-box 3 had a function in response to spotted leaf protein and was found to be orthologous with SlU-box 4.

**Fig. 10:**
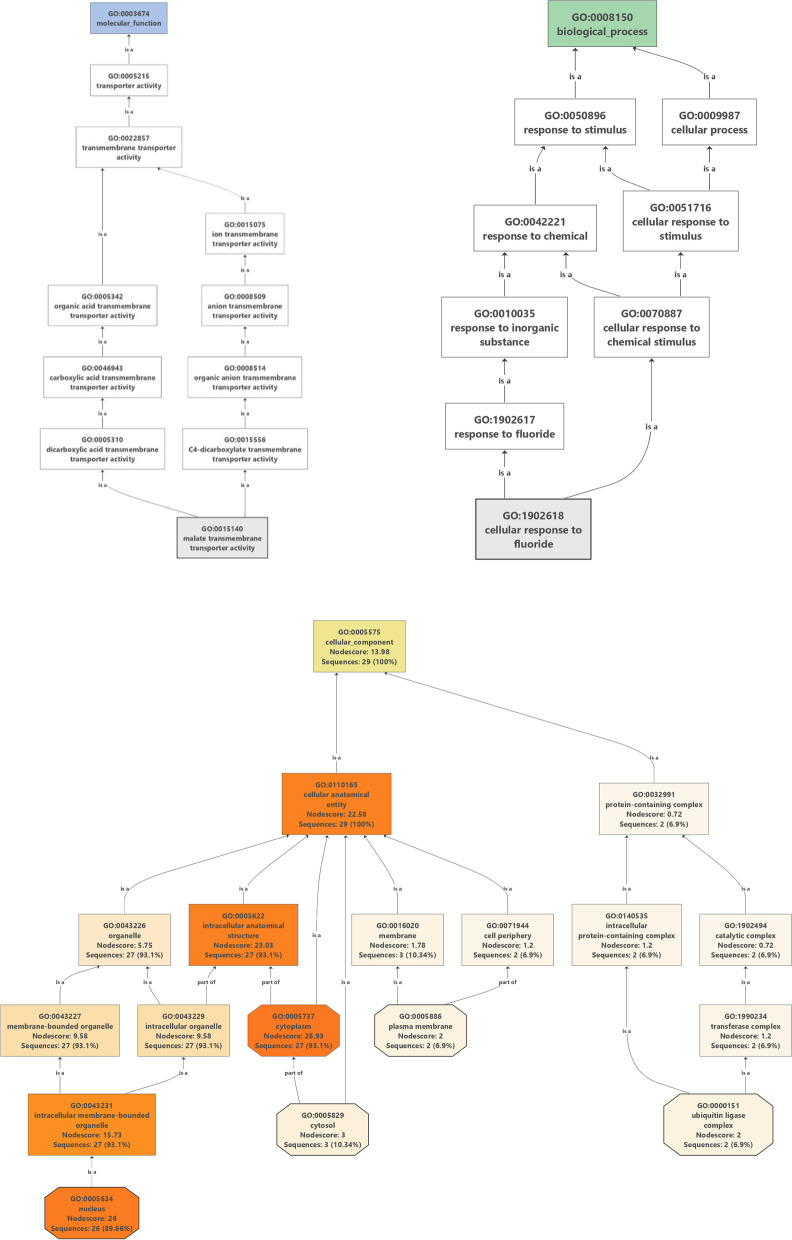
GO classification of the DEGs in potato. Percentage of the genes in cellular component, molecular function, and biological process classifications are displayed

The GO analysis revealed that the majority of the *StU*-*box* genes were involved in the response to stimuli, cellular response, response to chemical, cellular response to stimulus, and response to inorganic substance in biological processes. Further, more genes were implicated in transporter activity, transmembrane transporter activity, ion transmembrane transporter activity, and organic acid transmembrane transporter activity at levels of molecular function. In cellular component, most genes were involved in cellular, cellular anatomical entity, protein-containing complex, catalytic complex, intracellular protein-containing complex, cell periphery membrane, organelle, and intracellular anatomical (Fig. 10).

## Discussion

E3 ligases are an essential switcher of plant signaling paths that play through targeting proteins to the degradation path. These proteins constitute four separate subclasses, indicating that they are involved in various roles. In this study, our in silico analysis identified 62 potato *StU*-*box* genes. The detected *StU*-*box* genes were unevenly distributed on the 10 potato chromosomes. Features of the *StU*-*box* genes including peptide length, MW, Pi, and sub-cellular were analyzed. Our results agreed with previous studies in *Arabidopsis*, banana, grapevine, tomato, rice, cotton, and apple [9, 17, 24, 30, 37, 38, 42], StU-box proteins were mostly anticipated to be localized in nucleus, cytoplasm, and cell membrane. It is suggested that StU-boxs could function in the cytoplasm and nucleus-localized. In cotton, most of *GhU*-*box* genes are localized in the nucleus which agrees with our results in potato, consistent with their function as conserved gene [17]. In this study, GO analysis showed that majority of *U*-*box* genes are localized in membrane, organelle, cytoplasm, and cytosol. These results agreed with those reported in cotton and tomato [17, 24]. Further, most genes were involved in cellular processes and metabolic which was in agreement with results of Sharma and Taganna (2020).

The phylogenetic study of the potato *U*-*box* gene family revealed a great similarity among all the four classes due to the existence of the core U-box domain in all the members. The diversification of the *U*-*box* genes, whose members regulate key aspects of plant growth and development, is a clear example of the role that gene duplication and sub-functionalization play in shaping genetic systems. The sub-functionalization observed across the subfamily is required for the retention of family members in the genome. The augmentation of the gene family members could be a result of a neutral procedure of sub-functionalization. Together, these results indicate that sub-functionalization of expression has evolved relatively slowly. Sub-functionalization model predicts genomic features correlated with different expression profiles, phylogenetic and functional analyses, and the process of functional divergence of duplicated genes.

Gene duplication is a powerful mechanism providing the raw material for the evolution of the species and is the most common mechanism for the formation of original genes in these species [42]. Gene family expansion is associated with segmental and tandem duplications. Furthermore, whole-genome duplication, tandem, and segmental duplication have played key roles in the evolutionary expansion of gene families. The extension duplicated genes can also develop the acquisition of extended functions for the new genes. Tandem duplication tends to start modifications in gene structure and function more quickly than other mechanisms of duplication. StU-box 6 and 11 were involved in tandem duplication, suggesting that tandemly duplicated genes as a whole may play a vital role in signaling paths implicated in plant growth in potato. In *Eucalyptus grandis* and *A. thaliana*, expression analysis of paralogous gene pairs revealed differential expressions between paralogs in organs, supporting the notion that sub-functionalization and neo-functionalization occurred after duplication [7, 14, 15, 43].

Eighteen pairs of potato paralogs (StU-box 6 and StU-box 8, StU-box 6 and StU-box 9, StU-box 6 and StU-box 10, StU-box 6 and StU-box 11, StU-box 6 and StU-box 13, StU-box 8 and StU-box 9, StU-box 8 and StU-box 10, StU-box 8 and StU-box 11, StU-box 8 and StU-box 13, StU-box 9 and StU-box 10, StU-box 9 and StU-box 11, StU-box 9 and StU-box 13, StU-box 10 and StU-box 11, StU-box 10 and StU-box 13, StU-box 11 and StU-box 13, StU-box 17 and StU-box 46, StU-box 21 and StU-box 24, and StU-box 50 and StU-box 51) were involved in segmental duplications on different chromosomes. Four orthologous gene pairs of potato with *Arabidopsis* (StU-box 15 and AtU-box 37, StU-box 15 and AtU-box 55, StU-box 15 and AtU-box 58, and StU-box 27 and AtU-box 62), two orthologous gene pairs of potato with wheat (StU-box 27 and TaU-box 168, StU-box 15 and TaU-box 197), as well as three orthologous gene pairs of potato with rice (OsU-box 40 and StU-box 15, OsU-box 40 and StU-box 27, OsU-box 41 and StU-box 15) were identified. Tomato SlU-box 2 and SlU-box 3 were orthologous genes with potato StU-box 6, StU-box 9, StU-box 10, StU-box 11, and StU-box 13. Further, StU-box 3 with SlU-box 4 were orthologous. Three orthologous gene pairs of potato with maize (StU-box 15/30/31 and ZmU-box 44), 10 orthologous gene pairs of potato with cotton (StU-box 6/9/10/11/13 and GhU-box 41/68), as well as seven orthologous gene pairs of potato with citrus (StU-box 15 and CcU-box 14, StU-box 28 and CcU-box 26, StU-box 6/9/10/11/13 and CcU-box 29) were detected.

These results were the outcome of a putative tandem duplication occurrence. Our findings suggested that tandem gene duplication is the central cause of the expansion of the *U*-*box* gene family; similar findings have been reported in tomato and *Arabidopsis* [23]. Based on selective pressure analyses, most of the potato gene pairs were subjected to purify selection (negative) leading to removal of deleterious mutations. Also, our findings showed that *Arabidopsis* and citrus were exposed to purify selection. These findings are consistent with the outcomes reported for many other plant species [25]. However, in this survey, most genes in cotton, rice, tomato, wheat, and maize were considered as positive selection.

Gene duplication and syntenic study indicated that the segmental/tandem duplication are main forces for the diversity in the potato *U*-*box* genes. The syntenic analysis showed the structural and functional conservation of the genes, underlying the origins of the evolutionary novelty. Based on the evolutionary history of genes, orthologs have similar functions reflecting their conserved domains [2]. In the current survey, we found that *StU*-*boxs* could be functionally similar to their related homologs in *Arabidopsis*. The analysis separated the U-box proteins into four groups*. StU*-*box 15* was clustered with *AtU*-*box 37*, *AtU*-*box 55*, *AtU*-*box 58*, and *StU*-*box 27* was clustered with *AtU*-*box 62.* The *StU*-*box 15* gene was categorized into the class II, which included U-box and ARM domains. Further, StU-box 27 was categorized in the class IV including U-box, kinase, and USP domains. *U*-*box* genes with similar functions and structural domains revealed a trend to cluster in the same subfamilies. Genomic comparison with orthologous genes from well-studied plant species may provide a valuable reference for newly detected genes. Therefore, the functions of StU-box were inferred by comparative genomic analyses with the *U*-*box* gene from *Arabidopsis*. Four orthologous gene pairs between *Arabidopsis* and potato were detected, suggesting that these genes may share a common ancestor and their functions have been conserved during evolution. Although, supplementary investigation is required to examine the particular function of one gene.

Gene duplication plays a major factor in formation of domains, providing new opportunities to gain new gene functions for an organism. New domains may be illustrated by fusion, terminal domain loss, and duplication, likely driven by non-allelic homologous recombination, exon-shuffling, and transposon events. These kinds of rearrangements are overrepresented as duplicated genes, representing that these duplications influence the domain rearrangement rates. The arrangement and organization of the genes likewise show the diversity in a gene family among species. The organizational association is associated with the gene evolution and functional features of the gene family. Several *U*-*box* genes were either intron-less or with various introns. A parallel shape of intron-less genes of the *U*-*box* gene family was also reported in grape vine and tomato. The *U*-*box* genes bearing many introns could act as a mutational buffer, protecting coding sequences from randomly happening harmful mutations. The existence of the intron-less genes shows the organizational integrity among the members of U-box family. The distribution of the recognized 10 motifs among the tomato *U*-*box* gene family suggests the structural and functional identity among potato *U*-*box* genes. Motif 1 was found to be preserved and showed homology with the U-box domain. It likewise demonstrates the existence of further domains that may contribute to the critical structural construction in the *U*-*box* gene family. Motifs 2 and 3 were the limited features of the class II genes, resembling the armadillo-like fold structure.

Tissue expression profile analysis provided worth clues about the significant roles of *StU*-*box* genes for potato growth and developmental stages. For example, *StU*-*box 51* was exclusively expressed in tuber, root, stem, and leaf and *StU*-*box 27* was expressed in leaf. Four StU-boxes were approximately upregulated in the leaf. Although, StU-box 15 was upregulated in root. These data represented that several genes (e.g., the *StU*-*box* 51 and StU-box 27 gene) could play important roles in the development of leaf. This hypothesis strengthens the notion that they have important roles at leaf development stage in potato. In *Musa acuminate*, the *MaU*-*box* gene family had the highest expression in the roots [9]. *StU*-*box 51* was highly expressed in the tuber and leaf, indicating that it might be vital for tuber and leaf development. On the other hand, the expression levels of *StU*-*box 3*, *StU*-*box 15*, and *StU*-*box 27* was decreased during tuber, demonstrating that this gene might be involved in regulating leaf development.

Our findings indicate that the *U box* genes are found to be controlling several cellular processes namely root and shoot development, stolon growth, and tuber development. In *S. lycopersicum*, a high-rise in *U*-*box* gene expression was spotted in reproductive tissues, namely fruit and flower, suggesting the actions of U-box ligases in the critical plant development [24]. Qian et al. suggested that expression reduction, as a particular type of sub-functionalization, might assist the maintenance of duplicates and the conservation of their parental function [18].

The gene expression profiles of orthologus gene pairs, detected from syntenic analysis, were investigated to obtain understanding into functional consistency under different developmental steps and stress conditions. StU-box 3 was expressed in leaf. StU-box 3 is orthologous with SlU-box 4, where SlU-box 4 was expressed under heat shock conditions in the flower pollen tissue [24]. Further, SlU-box 4 was expressed in the leaf in tomato. However, StU-box 3 is downregulated under drought stress in potato. In *Arabidopsis*, AtPUB60 (At2g33340) and AtPUB49 (At5g67530) were highly expressed in leaf [32] which are similar to StU-box 3 (class I) in potato. These results can infer that abovementioned potato-tomato-*Arabidopsis* orthologs have similar functions. In rice, a spotted leaf gene *Spl7* encodes a heat shock protein and its mutation is responsible for lesion formation in the leaves [35].

Gene expression under drought stress indicated that StU-box 51 was upregulated in drought stress. AtPUB55 (*AT5G51270*) include Universal Stress Protein Domain (USPD in *Escherichia coli*) mediates survival under different stresses such as toxic chemicals, osmotic stress, UV light damage, and starvation to nutrients [12]. *At5g61560* (AtPUB58), orthologous StU-box 15, is a receptor-like protein kinase, has crucial regulatory roles in many aspects of plant growth and developmental. Further, this gene is involved in abiotic stress response namely, the abscisic acid response, calcium signaling, antioxidant defense, drought, salt, cold, and toxic metals/metalloids [36]. In this study, StU-box 15 is downregulated in drought. Our findings disagreed with previous studies in *Arabidopsis* and tomato [24, 32]. The concept of function of orthogous gene pairs could be destroyed by a subfunctionalization event which two orthologous gene pairs could be possess different functions.

StU-box 51 was expressed in root, stem, leaf, and tubers in class IV in potato, while class IV genes showed a weak expression profiles in most tissues. *StU*-*box 51* gene was upregulated, indicating that StU-box 51 contains many TFBS in its promoter region, including MYB, WRKY, bZIP, and NAC. MYB, bZIP, and NAC function in tuber development and play key roles in the upregulation of potato stolons. StU-box 3 and StU-box 27 were upregulated in leaf tissue due to a possible high number of MYB, bZIP, and WRKY. MYB and bZIP have been expressed under environmental stress and root storage [19, 20, 34]. WRKY had an important role in leaf tissues as well as was expressed in response to such stresses as wounding, drought, salt, and virus invasion [10, 21, 22]. StU-box 15 was upregulated in root tissues having a high number of DOF, AP2, and NAC. Dof is one of the most important TFs which was upregulated in root, shoot, leaf, and stolons. Based on the previous studies, some of *Dof* genes were expressed in all potato tissues while the expression levels of individual genes varied in different tissues [5, 21, 28]. In Brassica, AP2/ERF had specifically high expressions in the roots, although a few of the TFs were expressed in root and leaf [16, 40]. The profiles of genes expression confirm the *cis*-regulatory elements prediction making it even more lucid to culminate the community of the *U*-*box* gene family in the tomato development.

The regulatory mechanisms controlling *StU*-*box* gene expression were evaluated at transcriptional levels using TFBS in the promoter regions of *StU*-*box* genes. A total of 14,508 putative TFBS were involved in multiple biological processes. The extension of the gene family was observed as a course of evolution where gene duplication and sub-functionalization of the native U-box domain in higher eukaryotes played a major role. The Sub-functionalization is another tool that leads to the maintenance of duplicated genes while partitioning the ancestral function. The increase of the gene family members could be a result of a neutral procedure of sub-functionalization. Together, our findings suggest that sub-functionalization of expression evolves relatively slowly. To better insight why this is, we discovered which genomic features are correlated with divergent expression profiles of the duplicate genes.

According to the structure analysis results, motif identification, gene duplication, gene expression, syntenic analysis, analysis of TFBS, diverse members of the identical subfamily, and group had similar gene structure and conserved protein motifs, suggesting that they have similar evolutionary source and probably the same function. Orthologous genes are homologous genes reducing from a common ancestor separated by a speciation event, while paralogous genes are homologue genes generated by a duplication event. Often orthologous genes have an analogous function among various species, while paralogous genes have the same basic function, although they differ slightly in function. Thus, the survey of evolutionary genomics can shed light on the gene function. Genome-wide analysis of *U*-*box* genes showed that tandem duplication and chromosomal/segmental duplications possess important roles in *S*. *tuberosum* genome expansion. However, the number of tandem duplication was much higher than the number of segmental duplications, representing that these factors are the major components in the evolution of *U*-*box* genes. The characterization of *U*-*box* genes will provide a better understanding into their roles in several molecular functions. Our survey indicated the important participation of the *U*-*box* gene in the potato plant development. *U*-*box* gene is seldom surveyed in the potato plant system and our analysis provided an overall picture of the U-box gene family in potato. It will serve as an initial sign for the survey of the *U*-*box* genes in the potato and other plant systems.

## Conclusions

In this study, 62 *U*-*box* genes were recognized in *S*. *tuberosum*. Comprehensive analysis of *StU*-*box* genes were performed in terms of their chromosomal location, gene structure, gene expression profiles, analysis of TFBS, and evolutionary analysis. According to phylogenic relationships, StU-boxs were classified into four subfamilies, similar to the *U*-*box* genes in *Arabidopsis* and apple. The *U*-*box* genes might have apparently underwent gene loss and expansion through tandem duplication after polyploidization. All StU-boxs consisted U-box domains, whereas, some of them also had the ARM, Pkinase, and USP. Here, we observed that *StU*-*box 51* was highly expressed in root, stem, leaf, and tuber but was upregulated under drought. Therefore, StU-box 51 can be considered as a candidate gene in potato breeding programs. This study presents the first reported structural and functional analysis of *U*-*box* genes from *S*. *tuberosum.* This survey can provide a basis for further investigation of the regulation and functions of *StU*-*box* gene in growth and developmental stages of this important crop plant.

## Supplementary Information

**Additional file 1: Table S1.** Sequences and Pfam annotations of conserved motifs in StU-box proteins.

**Additional file 2: Table S2.** Summary of the Transcription factor binding sites (TFBS) detected in the promoter regions of *StU-box* genes.

**Additional file 3: Table S3.** Primer sequences used for real time RT-PCR in this study.

## Data Availability

Not applicable.

## Abbreviations

ARM: Armadillo
Pkinase_Tyr: Protein tyrosine kinase
PUB: Plant U-box
TFBS: Transcription factor binding sites
qRT-PCR: Quantitative real-time PCR
GO: Gene ontology
