# Supplementary material for: Genome-wide identification of StU-box gene family and assessment of their expression in developmental stages of Solanum tuberosum
Source: J Genet Eng Biotechnol. 2022 Feb 11;20:25. doi: 10.1186/s43141-022-00306-7 (PMC8837765; doi:10.1186/s43141-022-00306-7)
Supplement: Supplementary file 1 — Additional file 1: Table S1. Sequences and Pfam annotations of conserved motifs in StU-box proteins. [file 43141_2022_306_MOESM1_ESM.docx]

| Motif ID | Motif sequence | Pfam |
| --- | --- | --- |
| Motif 1 | IPDEFRCPISLELMK | [U-box](http://pfam.xfam.org/family/U-box), PF04564 |
| Motif 2 | AVAEJRLLAKRNADNRVVIAEAGAIPLLVNLLSSEDSSIQENAVTALLNLSINENNKKLI | [Arm](http://pfam.xfam.org/family/Arm) , [PF00514](http://pfam.xfam.org/family/PF00514.23) |
| Motif 3 | HGIERFPTPKPPVSKPQIIKLLKEAKSPKMQMKSLKRLRSIASENDANKRCMESAGAMEF | ARM, |
| Motif 4 | YMDPEYJDNGQFRPESDLYAFGIILLRJLAACPPNGJIRDFECAJESGNLGDVLDKSVGDWPJEZAEELAKLALQCCEKDCRDRP | [Pkinase_Tyr](http://pfam.xfam.org/family/Pkinase_Tyr), [PF07714](http://pfam.xfam.org/family/PF07714.17) |
| Motif 5 | DPVTISTGQTYERESIZKWJ | – |
| Motif 6 | LTHTDLTPNYTLRRLIQSWC | – |
| Motif 7 | EGSQRAKKDALTALFNLCPYGRNRVKAVEAGAVRVLVDLLL | – |
| Motif 8 | KRVCELMLILLDQLCTSAEGRAELLNHPGGLAIVSKKILRVSKVATERAIKILHSISKFS | – |
| Motif 9 | STPSVLQEMLSLGVVAKLCLVLQVDCGSKTKEKAREILKLHAKAWSNSPCIPNNLLSSYP | – |
| Motif 10 | IMSGNGEFIESLTRVMQHGSYESRAYAVMLMKDMFEVSTPTLLLSLKQEFFTQVVQVLRD | – |
