# Supplementary material for: Genome-wide identification of StU-box gene family and assessment of their expression in developmental stages of Solanum tuberosum
Source: J Genet Eng Biotechnol. 2022 Feb 11;20:25. doi: 10.1186/s43141-022-00306-7 (PMC8837765; doi:10.1186/s43141-022-00306-7)
Supplement: Supplementary file 2 — Additional file 2: Table S2. Summary of the Transcription factor binding sites (TFBS) detected in the promoter regions of StU-box genes. [file 43141_2022_306_MOESM2_ESM.docx]

|  |  |  |  |  |  |  |  |  |  |  |  |  |  |  |  |  |  |  |  |  |  |  |  |  |  |  |  |  |  |  |  |  |  |  |
| --- | --- | --- | --- | --- | --- | --- | --- | --- | --- | --- | --- | --- | --- | --- | --- | --- | --- | --- | --- | --- | --- | --- | --- | --- | --- | --- | --- | --- | --- | --- | --- | --- | --- | --- |
| Name of TFBS | Motifs/tfs related to tissue-specific localization | | | | | | | | Motifs/tfs related to hormone response | | | | | Motifs/tfs related to light response | | | Motifs/tfs related to basic transcription and expression levels | | | | Other tfs binding sites | | | | | | | | | Motifs/tfs related to stress response | | | | |
|  | AT-Hook | MADS box | MADF | SBP | LOB | WOX | TCR | HD-ZIP | AP2 | BES1 | ARF | EIN3 | VOZ | Dof | GATA | bHLH | NF-YB | Storekeeper | WRC | Sox | FAR1 | SRS | NAC | Homeodomain | bZIP | Trihelix | B3 | ZF-HD | TCP | C2H2 | MYB | HSF | WRKY | CSD |
| StU-box1 | 11 | 6 | 12 | 4 | 3 | 6 | 6 | 0 | 0 | 0 | 0 | 0 | 4 | 35 | 24 | 7 | 1 | 2 | 0 | 0 | 2 | 2 | 92 | 33 | 32 | 0 | 22 | 3 | 10 | 39 | 215 | 10 | 0 | 0 |
| StU-box2 | 15 | 11 | 17 | 27 | 3 | 5 | 5 | 38 | 138 | 11 | 4 | 3 | 8 | 47 | 14 | 51 | 1 | 2 | 1 | 0 | 4 | 2 | 64 | 38 | 65 | 22 | 19 | 2 | 30 | 33 | 132 | 1 | 87 | 1 |
| StU-box3 | 22 | 7 | 6 | 4 | 2 | 6 | 6 | 60 | 19 | 11 | 0 | 1 | 1 | 48 | 26 | 57 | 1 | 1 | 0 | 3 | 2 | 2 | 34 | 58 | 59 | 14 | 17 | 11 | 2 | 17 | 174 | 2 | 80 | 1 |
| StU-box4 | 18 | 11 | 5 | 1 | 0 | 5 | 1 | 54 | 10 | 0 | 2 | 5 | 0 | 53 | 19 | 11 | 1 | 0 | 1 | 2 | 0 | 2 | 33 | 51 | 27 | 10 | 13 | 2 | 1 | 25 | 83 | 1 | 24 | 0 |
| StU-box5 | 16 | 14 | 6 | 35 | 2 | 6 | 7 | 56 | 110 | 0 | 5 | 5 | 4 | 46 | 27 | 18 | 1 | 2 | 1 | 1 | 0 | 3 | 30 | 59 | 66 | 12 | 27 | 11 | 35 | 19 | 196 | 11 | 97 | 1 |
| StU-box6 | 15 | 5 | 1 | 4 | 0 | 4 | 6 | 35 | 7 | 0 | 0 | 0 | 3 | 17 | 25 | 12 | 1 | 1 | 1 | 1 | 1 | 1 | 12 | 38 | 26 | 4 | 7 | 2 | 4 | 32 | 109 | 1 | 55 | 0 |
| StU-box7 | 16 | 14 | 3 | 3 | 1 | 2 | 6 | 29 | 10 | 0 | 1 | 2 | 2 | 46 | 27 | 12 | 1 | 2 | 2 | 1 | 0 | 2 | 15 | 24 | 14 | 5 | 7 | 2 | 2 | 35 | 153 | 2 | 70 | 0 |
| StU-box8 | 14 | 5 | 5 | 4 | 1 | 5 | 4 | 39 | 6 | 11 | 0 | 1 | 3 | 22 | 23 | 56 | 2 | 1 | 1 | 0 | 1 | 1 | 16 | 39 | 52 | 8 | 9 | 2 | 3 | 32 | 121 | 1 | 55 | 0 |
| StU-box9 | 17 | 7 | 6 | 4 | 1 | 3 | 1 | 36 | 16 | 11 | 0 | 2 | 3 | 52 | 24 | 56 | 1 | 1 | 2 | 0 | 0 | 2 | 14 | 36 | 35 | 11 | 19 | 2 | 2 | 17 | 145 | 2 | 55 | 0 |
| StU-box10 | 9 | 2 | 7 | 3 | 1 | 3 | 5 | 36 | 18 | 0 | 1 | 1 | 0 | 41 | 23 | 11 | 1 | 0 | 1 | 0 | 3 | 1 | 17 | 37 | 22 | 11 | 18 | 2 | 1 | 13 | 120 | 2 | 20 | 1 |
| StU-box11 | # | 7 | 7 | 5 | 1 | 5 | 7 | 46 | 73 | 11 | 5 | 4 | 3 | 53 | 22 | 60 | 1 | 2 | 1 | 1 | 2 | 1 | 38 | 48 | 53 | 15 | 13 | 12 | 3 | 20 | 159 | 1 | 103 | 1 |
| StU-box12 | 9 | 2 | 7 | 3 | 1 | 3 | 5 | 36 | 18 | 0 | 1 | 1 | 0 | 41 | 23 | 11 | 1 | 0 | 1 | 0 | 3 | 1 | 17 | 37 | 22 | 11 | 18 | 2 | 1 | 13 | 120 | 2 | 20 | 1 |
| StU-box13 | 10 | 1 | 7 | 4 | 1 | 3 | 5 | 40 | 14 | 11 | 0 | 1 | 3 | 40 | 21 | 56 | 1 | 1 | 2 | 1 | 1 | 1 | 15 | 43 | 53 | 13 | 15 | 2 | 8 | 32 | 128 | 3 | 55 | 0 |
| StU-box14 | # | 5 | 13 | 36 | 2 | 7 | 4 | 9 | 51 | 0 | 2 | 1 | 3 | 30 | 23 | 16 | 1 | 2 | 2 | 3 | 1 | 2 | 38 | 60 | 42 | 19 | 18 | 11 | 7 | 40 | 163 | 2 | 102 | 0 |
| StU-box15 | # | 5 | 13 | 36 | 2 | 7 | 4 | 9 | 51 | 0 | 2 | 1 | 3 | 30 | 23 | 16 | 1 | 2 | 2 | 3 | 1 | 2 | 38 | 60 | 42 | 19 | 18 | 11 | 7 | 40 | 163 | 2 | 102 | 0 |
| StU-box16 | 16 | 16 | 6 | 20 | 2 | 6 | 4 | 58 | 22 | 0 | 3 | 6 | 3 | 52 | 20 | 18 | 1 | 1 | 2 | 3 | 0 | 2 | 18 | 61 | 55 | 13 | 22 | 11 | 6 | 33 | 167 | 11 | 99 | 0 |
| StU-box17 | 18 | 15 | 16 | 25 | 1 | 5 | 10 | 29 | 68 | 0 | 5 | 7 | 4 | 49 | 25 | 9 | 3 | 3 | 2 | 3 | 2 | 1 | 30 | 34 | 65 | 23 | 27 | 6 | 31 | 34 | 204 | 2 | 90 | 0 |
| StU-box18 | 15 | 4 | 10 | 4 | 0 | 4 | 4 | 38 | 29 | 0 | 3 | 4 | 4 | 48 | 5 | 13 | 1 | 1 | 0 | 2 | 0 | 2 | 46 | 40 | 59 | 17 | 16 | 2 | 30 | 38 | 162 | 2 | 82 | 0 |
| StU-box19 | 7 | 0 | 0 | 1 | 0 | 6 | 1 | 32 | 37 | 0 | 4 | 0 | 0 | 3 | 25 | 9 | 1 | 0 | 0 | 1 | 0 | 1 | 49 | 32 | 19 | 2 | 7 | 11 | 1 | 13 | 92 | 2 | 22 | 0 |
| StU-box20 | 15 | 6 | 1 | 5 | 1 | 1 | 5 | 45 | 19 | 0 | 1 | 4 | 4 | 50 | 11 | 13 | 1 | 1 | 0 | 0 | 0 | 2 | 10 | 42 | 33 | 4 | 21 | 1 | 1 | 28 | 131 | 0 | 75 | 1 |
| StU-box21 | 13 | 7 | 10 | 3 | 2 | 2 | 9 | 33 | 27 | 0 | 2 | 5 | 3 | 45 | 15 | 12 | 1 | 0 | 2 | 2 | 1 | 2 | 27 | 34 | 31 | 14 | 26 | 1 | 6 | 23 | 134 | 20 | 72 | 0 |
| StU-box22 | 19 | 7 | 10 | 36 | 3 | 3 | 6 | 57 | 102 | 0 | 5 | 1 | 5 | 56 | 27 | 15 | 1 | 0 | 2 | 0 | 1 | 3 | 81 | 57 | 56 | 14 | 27 | 5 | 26 | 40 | 145 | 3 | 106 | 1 |
| StU-box23 | 11 | 13 | 7 | 3 | 4 | 4 | 5 | 43 | 17 | 0 | 2 | 1 | 0 | 26 | 7 | 13 | 1 | 0 | 0 | 2 | 2 | 2 | 46 | 41 | 33 | 8 | 22 | 1 | 2 | 23 | 110 | 5 | 7 | 1 |
| StU-box24 | 7 | 9 | 14 | 8 | 3 | 2 | 3 | 49 | 43 | 0 | 1 | 1 | 4 | 51 | 12 | 13 | 1 | 1 | 2 | 3 | 0 | 1 | 35 | 48 | 94 | 20 | 16 | 11 | 3 | 24 | 158 | 20 | 86 | 1 |
| StU-box25 | 7 | 9 | 14 | 8 | 3 | 2 | 3 | 49 | 43 | 0 | 1 | 1 | 4 | 51 | 12 | 13 | 1 | 1 | 2 | 3 | 0 | 1 | 35 | 48 | 94 | 20 | 16 | 11 | 3 | 24 | 158 | 20 | 86 | 1 |
| StU-box26 | 13 | 10 | 5 | 28 | 0 | 3 | 3 | 28 | 15 | 0 | 2 | 3 | 0 | 48 | 24 | 15 | 3 | 1 | 1 | 1 | 0 | 2 | 41 | 32 | 31 | 8 | 18 | 4 | 2 | 35 | 137 | 5 | 25 | 0 |
| StU-box27 | 11 | 10 | 5 | 3 | 2 | 3 | 7 | 42 | 20 | 0 | 1 | 3 | 3 | 30 | 18 | 10 | 1 | 1 | 2 | 1 | 1 | 1 | 42 | 46 | 71 | 7 | 21 | 2 | 6 | 41 | 174 | 3 | 87 | 0 |
| StU-box28 | 10 | 10 | 1 | 23 | 0 | 6 | 8 | 0 | 11 | 0 | 1 | 0 | 0 | 16 | 25 | 7 | 1 | 0 | 1 | 1 | 0 | 1 | 14 | 38 | 37 | 4 | 13 | 8 | 2 | 28 | 120 | 5 | 3 | 0 |
| StU-box29 | 10 | 10 | 8 | 20 | 1 | 3 | 3 | 0 | 44 | 0 | 1 | 1 | 9 | 12 | 27 | 15 | 1 | 3 | 0 | 0 | 0 | 2 | 55 | 59 | 99 | 11 | 15 | 2 | 8 | 21 | 159 | 1 | 100 | 0 |
| StU-box30 | 2 | 1 | 1 | 3 | 2 | 1 | 4 | 16 | 147 | 0 | 5 | 1 | 1 | 11 | 21 | 14 | 1 | 0 | 0 | 2 | 0 | 1 | 3 | 20 | 25 | 5 | 15 | 3 | 15 | 21 | 87 | 1 | 21 | 1 |
| StU-box31 | 3 | 4 | 1 | 3 | 2 | 1 | 0 | 10 | 64 | 0 | 4 | 1 | 0 | 4 | 21 | 7 | 1 | 0 | 0 | 0 | 0 | 1 | 5 | 14 | 34 | 4 | 15 | 4 | 15 | 25 | 90 | 1 | 13 | 1 |
| StU-box32 | 19 | 13 | 7 | 28 | 5 | 6 | 9 | 47 | 94 | 1 | 6 | 4 | 6 | 53 | 23 | 17 | 1 | 2 | 1 | 3 | 4 | 2 | 48 | 45 | 47 | 13 | 28 | 10 | 4 | 38 | 200 | 7 | 93 | 0 |
| StU-box33 | 13 | 3 | 6 | 32 | 0 | 2 | 3 | 40 | 81 | 2 | 5 | 2 | 3 | 13 | 6 | 14 | 1 | 1 | 0 | 1 | 4 | 2 | 18 | 40 | 46 | 10 | 11 | 3 | 1 | 11 | 151 | 6 | 81 | 1 |
| StU-box34 | 10 | 16 | 9 | 33 | 1 | 4 | 4 | 32 | 30 | 1 | 5 | 3 | 3 | 23 | 26 | 16 | 1 | 2 | 2 | 1 | 0 | 3 | 41 | 34 | 69 | 16 | 17 | 7 | 14 | 22 | 165 | 3 | 68 | 0 |
| StU-box35 | 9 | 5 | 7 | 4 | 0 | 0 | 3 | 54 | 21 | 0 | 3 | 3 | 0 | 53 | 22 | 13 | 1 | 1 | 0 | 1 | 0 | 1 | 13 | 48 | 49 | 10 | 15 | 5 | 1 | 16 | 107 | 3 | 4 | 0 |
| StU-box36 | 11 | 7 | 10 | 30 | 0 | 4 | 12 | 33 | 18 | 0 | 2 | 2 | 1 | 55 | 28 | 12 | 1 | 1 | 0 | 0 | 1 | 2 | 22 | 33 | 20 | 15 | 18 | 1 | 4 | 39 | 156 | 2 | 24 | 0 |
| StU-box37 | 3 | 3 | 2 | 2 | 0 | 3 | 4 | 21 | 14 | 0 | 1 | 0 | 3 | 16 | 22 | 12 | 1 | 1 | 0 | 2 | 0 | 1 | 8 | 22 | 11 | 4 | 12 | 2 | 12 | 12 | 94 | 4 | 36 | 0 |
| StU-box38 | 10 | 3 | 12 | 35 | 0 | 4 | 5 | 39 | 20 | 11 | 2 | 5 | 0 | 50 | 27 | 59 | 1 | 1 | 2 | 0 | 1 | 2 | 33 | 39 | 36 | 17 | 20 | 2 | 8 | 18 | 169 | 3 | 18 | 0 |
| StU-box39 | 12 | 6 | 0 | 4 | 0 | 4 | 10 | 58 | 142 | 0 | 6 | 3 | 4 | 37 | 23 | 9 | 1 | 3 | 2 | 3 | 0 | 1 | 42 | 58 | 38 | 5 | 27 | 6 | 6 | 21 | 175 | 3 | 104 | 0 |
| StU-box40 | 9 | 18 | 6 | 34 | 2 | 6 | 6 | 29 | 140 | 11 | 0 | 3 | 3 | 31 | 8 | 64 | 2 | 2 | 1 | 0 | 0 | 1 | 28 | 26 | 48 | 9 | 19 | 1 | 3 | 14 | 176 | 1 | 105 | 1 |
| StU-box41 | 10 | 10 | 6 | 3 | 1 | 3 | 2 | 25 | 42 | 11 | 4 | 3 | 2 | 48 | 22 | 60 | 2 | 2 | 1 | 0 | 3 | 1 | 26 | 29 | 61 | 11 | 24 | 1 | 1 | 16 | 88 | 6 | 69 | 0 |
| StU-box42 | 14 | 13 | 9 | 24 | 2 | 7 | 0 | 16 | 43 | 1 | 2 | 2 | 0 | 42 | 25 | 11 | 1 | 3 | 2 | 1 | 4 | 2 | 19 | 22 | 64 | 14 | 15 | 10 | 2 | 31 | 154 | 15 | 105 | 0 |
| StU-box43 | 9 | 16 | 13 | 4 | 1 | 3 | 5 | 50 | 17 | 0 | 1 | 3 | 1 | 48 | 24 | 14 | 3 | 2 | 2 | 1 | 4 | 1 | 25 | 48 | 40 | 17 | 20 | 2 | 1 | 22 | 189 | 2 | 42 | 0 |
| StU-box44 | 17 | 7 | 2 | 26 | 1 | 3 | 5 | 47 | 80 | 0 | 5 | 4 | 3 | 47 | 20 | 14 | 1 | 2 | 1 | 0 | 0 | 2 | 27 | 48 | 27 | 4 | 22 | 4 | 5 | 26 | 124 | 3 | 99 | 0 |
| StU-box45 | 10 | 6 | 7 | 1 | 2 | 6 | 4 | 18 | 39 | 0 | 1 | 0 | 0 | 24 | 23 | 12 | 2 | 1 | 2 | 2 | 0 | 2 | 14 | 24 | 30 | 9 | 23 | 5 | 2 | 15 | 76 | 0 | 18 | 0 |
| StU-box46 | 15 | 15 | 11 | 24 | 2 | 5 | 6 | 21 | 134 | 0 | 1 | 6 | 4 | 38 | 25 | 12 | 3 | 2 | 2 | 3 | 0 | 1 | 24 | 29 | 32 | 18 | 20 | 3 | 9 | 32 | 168 | 2 | 103 | 0 |
| StU-box47 | # | 5 | 1 | 4 | 0 | 6 | 3 | 29 | 20 | 0 | 1 | 1 | 0 | 32 | 20 | 15 | 1 | 0 | 0 | 2 | 0 | 1 | 22 | 28 | 38 | 8 | 23 | 9 | 2 | 24 | 91 | 2 | 19 | 1 |
| StU-box48 | 9 | 11 | 10 | 5 | 2 | 4 | 2 | 22 | 144 | 11 | 5 | 4 | 3 | 42 | 18 | 63 | 3 | 3 | 1 | 0 | 1 | 2 | 29 | 29 | 33 | 12 | 22 | 1 | 15 | 15 | 114 | 2 | 97 | 0 |
| StU-box49 | 7 | 4 | 2 | 12 | 0 | 3 | 6 | 51 | 49 | 0 | 6 | 1 | 8 | 48 | 21 | 17 | 2 | 1 | 2 | 3 | 0 | 1 | 24 | 51 | 42 | 8 | 19 | 1 | 3 | 15 | 124 | 2 | 96 | 0 |
| StU-box50 | # | 26 | 11 | 25 | 1 | 6 | 10 | 65 | 29 | 1 | 3 | 6 | 4 | 55 | 20 | 21 | 1 | 1 | 2 | 3 | 3 | 2 | 60 | 64 | 58 | 16 | 28 | 8 | 18 | 44 | 219 | 5 | 105 | 1 |
| StU-box51 | 13 | 19 | 9 | 25 | 1 | 5 | 5 | 42 | 20 | 1 | 2 | 4 | 4 | 39 | 20 | 21 | 1 | 1 | 2 | 1 | 0 | 2 | 47 | 46 | 47 | 13 | 23 | 6 | 12 | 15 | 159 | 1 | 103 | 0 |
| StU-box52 | 11 | 7 | 4 | 4 | 3 | 4 | 5 | 21 | 28 | 1 | 2 | 5 | 1 | 46 | 24 | 15 | 1 | 1 | 2 | 2 | 0 | 1 | 40 | 24 | 39 | 7 | 22 | 1 | 0 | 18 | 125 | 16 | 25 | 0 |
| StU-box53 | 7 | 20 | 6 | 20 | 2 | 5 | 9 | 29 | 30 | 11 | 2 | 6 | 9 | 53 | 25 | 59 | 1 | 0 | 2 | 3 | 2 | 2 | 75 | 34 | 87 | 13 | 23 | 11 | 14 | 21 | 191 | 11 | 100 | 0 |
| StU-box54 | 10 | 23 | 8 | 15 | 3 | 8 | 4 | 60 | 70 | 0 | 5 | 3 | 0 | 14 | 23 | 15 | 1 | 1 | 2 | 1 | 0 | 2 | 18 | 61 | 44 | 12 | 24 | 9 | 3 | 27 | 137 | 9 | 92 | 0 |
| StU-box55 | 13 | 1 | 7 | 3 | 0 | 3 | 4 | 29 | 15 | 0 | 3 | 2 | 5 | 48 | 14 | 21 | 1 | 0 | 1 | 0 | 0 | 1 | 36 | 32 | 34 | 13 | 14 | 1 | 1 | 12 | 140 | 5 | 76 | 0 |
| StU-box56 | 9 | 4 | 3 | 3 | 0 | 3 | 2 | 52 | 5 | 1 | 0 | 3 | 0 | 32 | 2 | 13 | 2 | 0 | 1 | 0 | 0 | 1 | 13 | 48 | 86 | 5 | 4 | 1 | 0 | 26 | 116 | 2 | 21 | 0 |
| StU-box57 | 13 | 3 | 11 | 16 | 5 | 6 | 4 | 19 | 56 | 0 | 2 | 2 | 1 | 46 | 29 | 9 | 1 | 3 | 1 | 1 | 0 | 2 | 14 | 21 | 14 | 14 | 17 | 10 | 3 | 19 | 195 | 1 | 11 | 1 |
| StU-box58 | 11 | 23 | 9 | 4 | 1 | 6 | 6 | 17 | 22 | 11 | 3 | 4 | 5 | 49 | 17 | 61 | 3 | 1 | 1 | 1 | 2 | 1 | 40 | 23 | 36 | 15 | 20 | 2 | 14 | 20 | 146 | 3 | 74 | 0 |
| StU-box59 | 17 | 14 | 4 | 4 | 2 | 4 | 6 | 20 | 20 | 0 | 1 | 2 | 0 | 51 | 20 | 12 | 2 | 1 | 0 | 1 | 4 | 2 | 16 | 26 | 23 | 7 | 19 | 1 | 2 | 38 | 119 | 2 | 11 | 1 |
| StU-box60 | 15 | 14 | 9 | 32 | 3 | 2 | 5 | 44 | 64 | 0 | 2 | 3 | 4 | 26 | 29 | 14 | 3 | 2 | 1 | 4 | 2 | 1 | 41 | 47 | 44 | 12 | 22 | 3 | 5 | 14 | 118 | 2 | 78 | 0 |
| StU-box61 | 5 | 7 | 4 | 3 | 1 | 3 | 3 | 46 | 14 | 0 | 2 | 6 | 0 | 47 | 25 | 13 | 2 | 2 | 0 | 2 | 2 | 2 | 60 | 46 | 43 | 5 | 14 | 3 | 4 | 17 | 114 | 2 | 3 | 0 |
| StU-box62 | 9 | 2 | 6 | 26 | 1 | 2 | 7 | 39 | 26 | 0 | 1 | 3 | 6 | 39 | 17 | 12 | 2 | 1 | 1 | 3 | 0 | 2 | 54 | 43 | 41 | 8 | 18 | 2 | 4 | 36 | 146 | 2 | 31 | 1 |
