# Supplementary material for: Genome-wide identification of StU-box gene family and assessment of their expression in developmental stages of Solanum tuberosum
Source: J Genet Eng Biotechnol. 2022 Feb 11;20:25. doi: 10.1186/s43141-022-00306-7 (PMC8837765; doi:10.1186/s43141-022-00306-7)
Supplement: Supplementary file 3 — Additional file 3: Table S3. Primer sequences used for real time RT-PCR in this study. [file 43141_2022_306_MOESM3_ESM.docx]

| **No.** | Name gene | Sequence primer |
| --- | --- | --- |
| **1** | F:EF1α  R:EF1α | AGATGGTCAGACCCGTGAAC  CCTTGGAGTACTTCGGGGTG |
| **2** | F:StU-box3  R:StU-box3 | TCAGCAACAAGTGGAGTTC  CATTAAGCATACCTTGTTAAGGTG |
| **3** | F:StU-box15  R:StU-box15 | TCCGAAGAGCATTGTGACATCC  TGTCAATTCTGTTGCAGCCACTCC |
| **4** | F:StU-box27  R:StU-box27 | TCAGGATGTCATGGAAGATCCAC  TGAGTTTCAGAACTGTTGCTGCC |
| **5** | F:StU-box51  R:StU-box51 | TCCACATTAGACGTTCGCAC  ACTTCAACTCAGTCTCCAAGCC |
